# SGLT2 Inhibition Combined with Zn Supplementation Protects the Liver-Pancreas Axis in Experimental Type 2 Diabetes

**DOI:** 10.3390/ijms27209067

**Published:** 2026-10-13

**Authors:** Irina Claudia Anton, Carmen Solcan, Liliana Mititelu-Tartau, Cornelia Amalinei, Mihaela Poroch, Vladimir Poroch, Beatrice Rozalina Buca, Cosmin-Gabriel Tartau, Ana-Maria Pelin, Alexandra Jitareanu, Mihail Virgil Boldeanu, Gina Eosefina Botnariu

**Affiliations:** 1 Grigore T. Popa, University of Medicine and Pharmacy, Universitatii St. 16, 700115 Iasi, Romania; irinaanton@taktfest.ro (I.C.A.); liliana.tartau@umfiasi.ro (L.M.-T.); cornelia.amalinei@umfiasi.ro (C.A.); boanca.mihaela@umfiasi.ro (M.P.); vladimir.poroch@umfiasi.ro (V.P.); beatrice-rozalina.buca@umfiasi.ro (B.R.B.); cosmin.tartau@gmail.com (C.-G.T.); jitareanu.alexandra@umfiasi.ro (A.J.); ginabotnariu66@gmail.com (G.E.B.); 2 Ion Ionescu de la Brad University of Life Sciences, Aleea Mihail Sadoveanu No. 3, 700490 Iasi, Romania; 3 Dunarea de Jos University, 800010 Galati, Romania; anapelin@gmail.com; 4 University of Medicine and Pharmacy Craiova, 200349 Craiova, Romania; mihail.boldeanu@umfcv.ro

**Keywords:** type 2 diabetes mellitus, SGLT2 inhibitors, DAPA, EMPA, Zn supplementation, pancreatic β-cells, hepatic injury

## Abstract

Type 2 diabetes mellitus (T2DM) is associated with chronic hyperglycemia, inflammation, and progressive hepatic and pancreatic dysfunction. Although sodium-glucose cotransporter 2 (SGLT2) inhibitors exert metabolic and organ-protective effects, their ability to preserve pancreatic architecture and attenuate diabetes-associated hepatic injury, particularly combined with nutritional interventions, remains insufficiently explored. This study investigated the protective effects of dapagliflozin (DAPA) and empagliflozin (EMPA), alone or combined with zinc (Zn), on hepatic and pancreatic alterations in experimental diabetes. Methods: Diabetes was induced in male Sprague–Dawley rats using streptozotocin (STZ) and high fat diet (HFD). Diabetic animals received DAPA, EMPA, Zn, or SGLT2 inhibitor–Zn combinations for four weeks. Hepatic biochemical parameters, lipid profile, histopathology, pancreatic morphometry, and insulin immunoreactivity were evaluated. Diabetes induced hepatic and pancreatic alterations, including elevated liver injury markers, hepatocellular degeneration, reduced islet number and β-cell density, and diminished insulin immunoreactivity. DAPA and EMPA attenuated these changes, with greater improvements following Zn combination therapy. EMPA + Zn produced the most favorable response, improving hepatic architecture, reducing VCAM-1, ICAM-1, and NF-κB expression, preserving islet morphology, increasing β-cell density, and enhancing insulin immunoreactivity. SGLT2 inhibitors attenuated diabetes-associated hepatic and pancreatic alterations in this experimental model, while Zn supplementation was associated with additional tissue-preserving effects, particularly when combined with EMPA. These findings support further investigation of combined SGLT2 inhibition and Zn supplementation as an experimental approach to diabetes-associated organ injury; however, their clinical relevance remains to be established.

## 1. Introduction

### 1.1. Type 2 Diabetes Mellitus and the Liver–Pancreas Axis

T2DM is a major global metabolic disorder whose prevalence continues to increase in association with population aging, sedentary lifestyle, and obesity [1,2]. Although chronic hyperglycemia is a defining feature, T2DM is a systemic disorder characterized by insulin resistance, impaired glucose and lipid metabolism, chronic inflammation, and progressive tissue dysfunction [3,4]. The development and progression of T2DM are driven by the interaction between insulin resistance and pancreatic β-cell dysfunction. Initially, β-cells compensate for reduced insulin sensitivity by increasing insulin secretion; however, persistent exposure to hyperglycemia, excess lipids, inflammatory mediators, and cellular stress progressively impairs β-cell function and metabolic control [5,6,7].

Increasing evidence supports the concept that the liver and pancreas constitute an interconnected metabolic axis involved in T2DM progression [8,9,10]. Under physiological conditions, pancreatic insulin regulates hepatic glucose production and lipid metabolism, while hepatic metabolic signals contribute to the regulation of pancreatic endocrine function [11]. In T2DM, disruption of this bidirectional communication promotes excessive hepatic glucose production, lipid accumulation, inflammation, and progressive pancreatic β-cell dysfunction, thereby establishing a self-perpetuating cycle of metabolic deterioration [12,13].

The liver is particularly susceptible to insulin resistance and metabolic overload, with impaired insulin signaling promoting hepatic glucose production and triglyceride (TG) accumulation and contributing to metabolic dysfunction-associated steatotic liver disease (MASLD) [14]. MASLD is highly prevalent in individuals with T2DM and is associated with steatohepatitis, fibrosis, and increased cardiometabolic risk [15,16,17]. At the same time, pancreatic β-cells are vulnerable to glucotoxicity, lipotoxicity, oxidative and cellular stress, which can promote mitochondrial dysfunction, apoptosis, and progressive loss of endocrine function [18,19]. Thus, hepatic and pancreatic injury should be considered interconnected manifestations of the same metabolic disorder rather than isolated complications [5,20].

### 1.2. Interconnected Mechanisms of Hepatic and Pancreatic Injury

The metabolic and inflammatory disturbances underlying T2DM contribute simultaneously to hepatic and pancreatic injury [8,21]. Persistent hyperglycemia and lipid excess promote oxidative stress, mitochondrial dysfunction, and inflammatory signaling, while glucolipotoxicity directly contributes to β-cell dysfunction and hepatic lipid accumulation [22,23,24,25,26,27,28,29,30,31]. Activation of inflammatory pathways, including nuclear factor-kappa B (NF-κB), together with increased expression of adhesion molecules such as intercellular adhesion molecule-1 (ICAM-1) and vascular cell adhesion molecule-1 (VCAM-1), further contributes to tissue injury and remodeling [32,33,34,35].

These mechanisms are relevant to both the liver and pancreas and provide a rationale for therapeutic approaches that extend beyond glucose lowering to preserve tissue integrity and modulate diabetes-associated inflammation [36,37]. Accordingly, simultaneous assessment of hepatic and pancreatic responses may provide a more integrated understanding of therapeutic protection in experimental T2DM.

### 1.3. SGLT2 Inhibitors as Potential Organ-Protective Agents

SGLT2 inhibitors are established treatments for T2DM that reduce renal glucose reabsorption through an insulin-independent mechanism [38,39]. Their clinical benefits extend beyond glycemic control and include cardiovascular and renal protection, supporting the concept that these agents exert broader metabolic and tissue-protective effects [40,41,42,43,44].

Experimental and clinical evidence also suggests that SGLT2 inhibitors may influence organs directly affected by diabetes. In the liver, agents such as DAPA and EMPA have been associated with reduced lipid accumulation and attenuation of inflammatory and metabolic disturbances relevant to MASLD [45,46]. In the pancreas, SGLT2 inhibition has been suggested to preserve β-cell integrity and insulin-producing capacity, potentially by reducing the metabolic burden associated with chronic hyperglycemia [47,48]. However, because hepatic and pancreatic injury involves multiple interacting mechanisms, improvement of glycemia alone may not fully prevent tissue damage, providing a rationale for complementary therapeutic strategies [49].

### 1.4. Zinc as a Potential Adjuvant to SGLT2 Inhibition

Zn is an essential trace element involved in insulin synthesis, storage and secretion, cellular metabolism, immune regulation, and maintenance of tissue homeostasis [50,51,52,53]. Diabetes may disrupt Zn homeostasis through altered metabolism, increased urinary losses, and chronic inflammation, potentially contributing to impaired insulin signaling and β-cell dysfunction [52,54,55].

The pancreas is particularly relevant to Zn biology because β-cells require Zn for insulin maturation, storage, and secretion. Experimental studies indicate that Zn deficiency can impair β-cell function, whereas Zn supplementation may preserve islet architecture and insulin-producing capacity in diabetic models [56,57,58,59,60]. Zn also participates in cellular defense and inflammatory regulation, including modulation of NF-κB signaling and cytokine production [61,62]. In the liver, Zn may contribute to the maintenance of cellular integrity and regulation of inflammatory responses during metabolic stress [61,63].

Importantly, several mechanisms potentially influenced by Zn—including metabolic regulation, inflammatory control, and preservation of cellular homeostasis—overlap with mechanisms proposed for the organ-protective effects of SGLT2 inhibitors [60,64,65]. This overlap provides a mechanistic rationale for investigating Zn as an adjunct to SGLT2 inhibition. Nevertheless, the combined effects of Zn supplementation with DAPA or EMPA on the liver–pancreas axis in experimental T2DM remain insufficiently characterized.

### 1.5. Knowledge Gap, Study Rationale and Objectives

Although SGLT2 inhibitors have demonstrated metabolic, cardiovascular, renal, and potential organ-protective effects, diabetes-associated hepatic and pancreatic injury may persist despite therapeutic intervention [66,67,68,69]. Moreover, most previous studies have evaluated individual target organs or investigated SGLT2 inhibitors and Zn supplementation separately. The potential of Zn to complement SGLT2 inhibition and provide coordinated protection of both hepatic and pancreatic tissues has therefore not been sufficiently investigated.

The present study addressed this gap by evaluating the effects of DAPA and EMPA, administered alone or in combination with Zn supplementation, in a HFD/STZ-induced rat model of T2DM. Rather than focusing exclusively on glycemic control, we examined the liver–pancreas axis using an integrated biochemical, histopathological, morphometric, and immunohistochemical approach. Hepatic injury and tissue alterations were evaluated together with inflammatory signaling markers (NF-κB, ICAM-1, and VCAM-1), while pancreatic islet morphology, β-cell preservation, and insulin immunoreactivity were assessed as indicators of endocrine pancreatic integrity.

The novelty of the present study lies in the integrated evaluation of hepatic and pancreatic tissue protection following DAPA or EMPA treatment, with or without Zn supplementation, thereby addressing whether Zn may provide complementary protection beyond the effects of SGLT2 inhibition alone. We hypothesized that Zn supplementation would provide additional tissue-protective effects when combined with SGLT2 inhibitors through complementary modulation of inflammatory and cellular injury pathways, potentially contributing to the preservation of liver–pancreas integrity in experimental T2DM.

## 2. Results

Successful induction of diabetes was confirmed by the marked increase in fasting blood glucose concentrations following STZ administration. Metabolic parameters from the same experimental cohort have been previously reported in detail [60]. To provide the metabolic context necessary for the interpretation of the hepatic and pancreatic findings presented in the current study, fasting blood glucose and body weight data at baseline and at the end of the experimental period are summarized in Table 1.

The previously reported data showed that STZ administration resulted in a marked increase in fasting blood glucose, whereas treatment with DAPA or EMPA, administered alone or in combination with Zn supplementation, was associated with improved glycemic control [60]. Body weight increased during the experimental period in all groups, although the magnitude of weight gain differed among experimental groups [60]. These metabolic parameters are therefore presented alongside the hepatic and pancreatic outcomes to facilitate interpretation of the observed tissue-protective effects in the context of the overall metabolic response to treatment.

### 2.1. Serum Biochemical Markers of Hepatic Function and Tissue Injury

**Figure 1:**
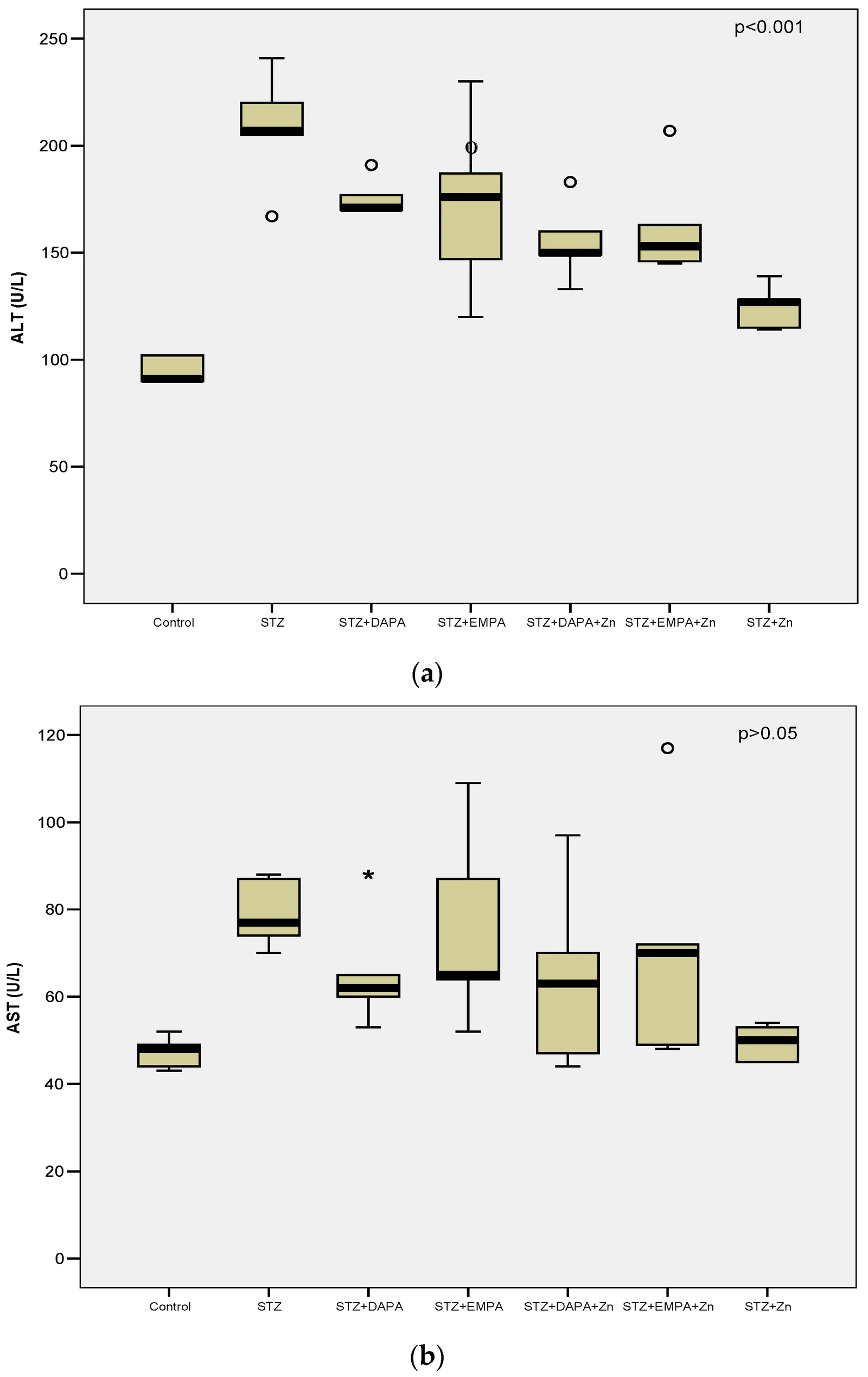
The modifications of (**a**) ALT and (**b**) AST serum levels. Data are presented as box-and-whisker plots, with the horizontal line within each box representing the median and the box representing the interquartile range (IQR). Whiskers indicate the range of non-outlier values. Circles represent outliers (values between 1.5 and 3 × IQR from the quartiles), whereas asterisks represent extreme values (values > 3 × IQR from the quartiles). Statistical significance between groups is indicated in the respective panels (n = 5 animals per group).

Serum alanin aminotransferase (ALT) activity ranged from 90 to 241 U/L and differed significantly among the experimental groups (*p* = 0.001; Figure 1a). The Control group exhibited the lowest mean ALT activity (95.0 ± 6.40 U/L), whereas STZ rats showed the highest value (208.0 ± 27.04 U/L). The difference between these groups was associated with an extremely large standardized effect (Hedges’ g = 5.19), confirming the marked hepatocellular injury induced by STZ. All therapeutic interventions reduced ALT activity relative to untreated diabetic animals, with the greatest improvement observed in the STZ + Zn group (124.60 ± 10.36 U/L), followed by the STZ + DAPA + Zn group (155.00 ± 21.94 U/L), whereas DAPA and EMPA monotherapy produced more modest reductions (Figure 1a, Table S1—Supplementary Files).

Aspartate aminotransferase (AST) activity showed a variation across the experimental groups, with values ranging from 43 to 117 U/L (*p* > 0.05; Figure 1b). The untreated diabetic group displayed a marked elevation in AST activity compared with the Control group, reaching a mean value of 79.20 ± 7.98 U/L versus 47.20 ± 3.70 U/L, respectively. This difference was associated with an extremely large standardized effect size (Hedges’ g = 4.64), indicating a pronounced alteration in hepatic enzyme activity in the diabetic condition. Among the interventions, Zn supplementation resulted in the most evident normalization of AST activity, with values approaching those observed in the Control group (49.40 ± 4.29 U/L). DAPA was associated with a moderate decrease in AST activity, whereas EMPA produced a comparatively modest change (Figure 1b, Table S1—Supplementary Files).

**Figure 2:**
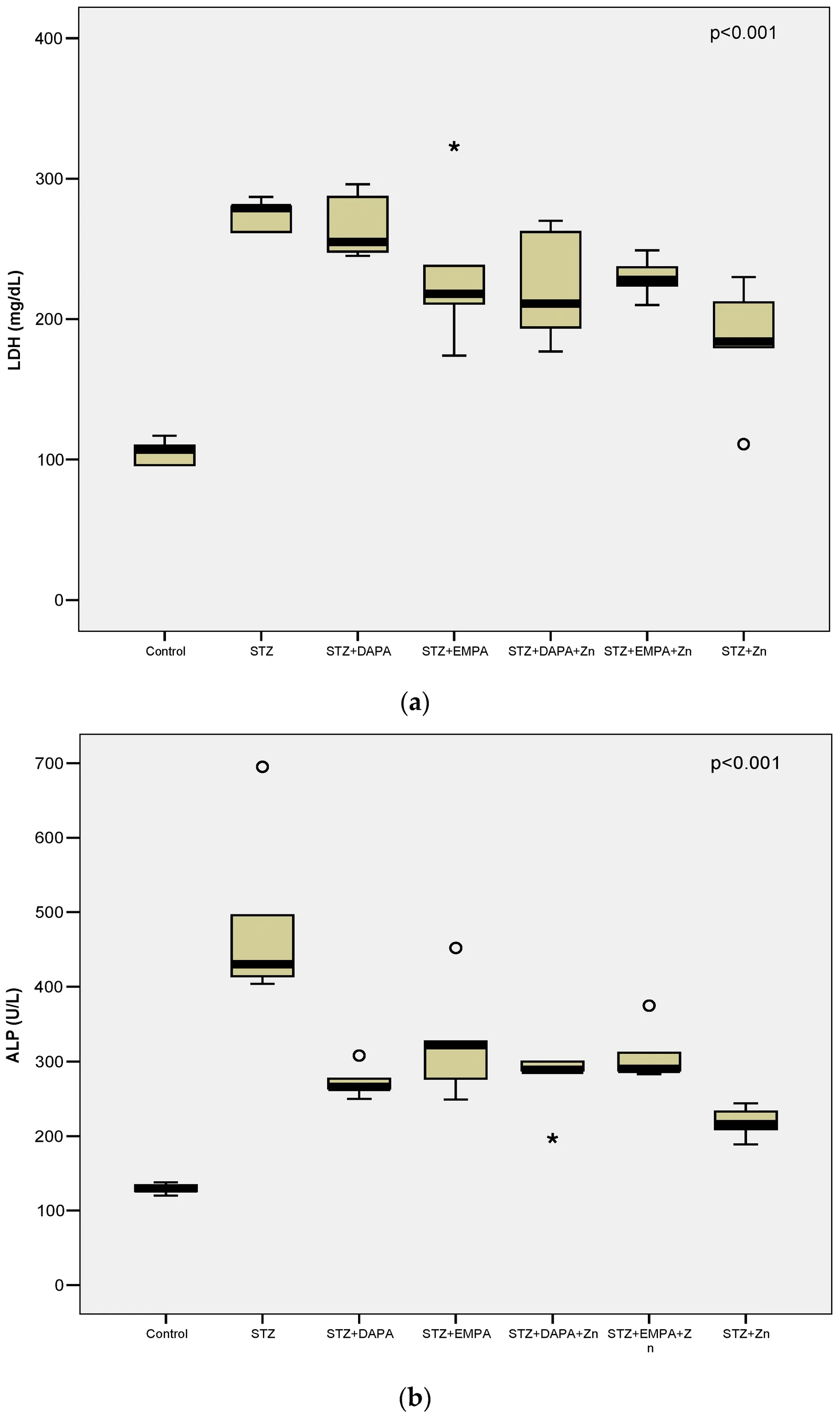
The modifications of (**a**) LDH and (**b**) ALP serum levels. Data are presented as box-and-whisker plots, with the horizontal line within each box representing the median and the box representing the interquartile range (IQR). Whiskers indicate the range of non-outlier values. Circles represent outliers (values between 1.5 and 3 × IQR from the quartiles), whereas asterisks represent extreme values (values > 3 × IQR from the quartiles). Statistical significance between groups is indicated in the respective panels (n = 5 animals per group).

Serum lactate dehydrogenase (LDH) levels varied substantially across the experimental groups, ranging from 96 to 323 mg/dL, with a statistically significant overall difference (*p* = 0.001; Figure 2a). The lowest LDH value was recorded in the Control group (105.20 ± 9.15 mg/dL), while markedly higher concentrations were observed in STZ-treated animals (274.00 ± 11.38 mg/dL). The contrast between these groups yielded an extremely large standardized effect size (Hedges’ g = 14.68), highlighting the pronounced tissue damage associated with STZ-induced diabetes. Treatment-related differences were also evident: Zn supplementation produced the strongest reduction in LDH, resulting in a mean value of 183.40 ± 45.40 mg/dL in the STZ + Zn group. A reduction was likewise observed following combined DAPA + Zn treatment (222.80 ± 41.32 mg/dL), whereas DAPA alone exerted a comparatively modest effect (Figure 2a, Table S2—Supplementary Files).

A marked between-group variation was observed for alkaline phosphatase (ALP), with values ranging from 120 to 695 U/L (*p* = 0.001; Figure 2b). The untreated diabetic animals had substantially higher ALP levels than the Control group (487.80 ± 121.25 vs. 129.60 ± 6.99 U/L), corresponding to an extremely large standardized effect (Hedges’ g = 3.76) and indicating pronounced hepatobiliary impairment associated with diabetes. Treatment was associated with a clear attenuation of this increase, most notably in the STZ + Zn group, where ALP reached 218.20 ± 21.37 U/L. DAPA alone resulted in a mean value of 273.00 ± 21.79 U/L, while the addition of Zn to DAPA produced a similar level (274.80 ± 43.87 U/L) (Figure 2b, Table S2—Supplementary Files). Overall, Zn supplementation produced the greatest normalization of ALP among the investigated interventions.

### 2.2. Lipid Profile

**Figure 3:**
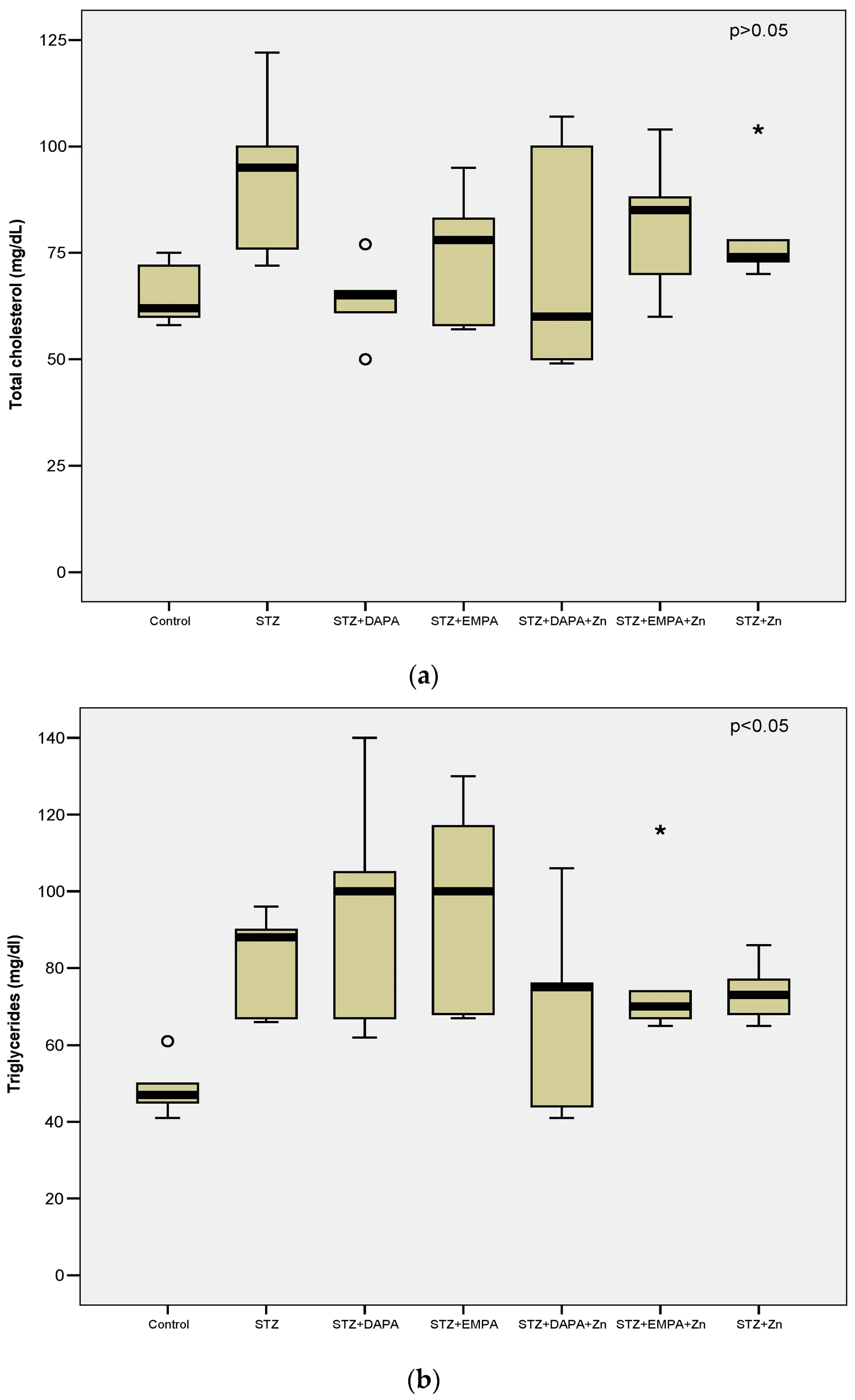
The modifications of (**a**) total cholesterol and (**b**) triglycerides serum levels. Data are presented as box-and-whisker plots, with the horizontal line within each box representing the median and the box representing the interquartile range (IQR). Whiskers indicate the range of non-outlier values. Circles represent outliers (values between 1.5 and 3 × IQR from the quartiles), whereas asterisks represent extreme values (values > 3 × IQR from the quartiles). Statistical significance between groups is indicated in the respective panels (n = 5 animals per group).

The blood level of total cholesterol varied between 49 and 122 mg/dL across the experimental groups, without reaching statistical significance (*p* = 0.163; Figure 3a). The mean concentration was 65.40 ± 7.60 mg/dL in the Control group and increased to 93.00 ± 20.15 mg/dL in untreated diabetic rats, representing the highest group mean. Among the intervention groups, the STZ + DAPA group showed the lowest mean cholesterol level (63.80 ± 9.73 mg/dL), whereas the highest value was observed following combined EMPA + Zn treatment (81.40 ± 16.99 mg/dL). However, the observed numerical variation was not accompanied by statistically significant between-group differences. These findings suggest that, under the experimental conditions of the present study, neither diabetes induction nor the tested treatment regimens produced a clear alteration in total cholesterol homeostasis (Figure 3a, Table S3—Supplementary Files).

Triglyceride (TG) levels ranged from 41 to 140 mg/dL across the experimental groups (Figure 3b). The Control animals exhibited the lowest mean concentration (48.80 ± 7.56 mg/dL), whereas the highest values were recorded in the STZ + EMPA (96.40 ± 28.45 mg/dL) and STZ + DAPA (94.80 ± 31.71 mg/dL) groups. Pairwise analysis revealed significantly higher TG concentrations in both SGLT2 inhibitor monotherapy groups compared with the Control group, with statistical significance observed for STZ + DAPA (*p* = 0.049) and STZ + EMPA (*p* = 0.029) (Figure 3b). By contrast, the groups receiving Zn in combination with SGLT2 inhibitors did not differ significantly from the untreated diabetic group. This pattern suggests that Zn supplementation may have partially attenuated the increase in circulating TG associated with DAPA and EMPA monotherapy (Figure 3b, Table S3—Supplementary Files).

Neither LDL-cholesterol (LDL-C) nor HDL-cholesterol (HDL-C) demonstrated significant between-group variation, with both parameters showing only modest fluctuations across the experimental conditions (*p* > 0.05). At T3, HDL-C concentrations ranged from 11.19 to 38.38 mg/dL across the study population. No statistically significant differences were observed in mean HDL-C levels among the study groups (*p* = 0.114). The lowest mean HDL-C concentration was recorded in Control, with a value of 16.47 ± 0.41 mg/dL, whereas the highest mean concentration was observed in group which received combined EMPA + Zn treatment, at 27.94 ± 9.08 mg/dL. The assessment of LDL-C at T3 revealed values ranging between 4.47 and 11.83 mg/dL. Comparison of the study groups showed no statistically significant variation in mean LDL-C concentrations (*p* = 0.412). Group III, receiving DAPA treatment, exhibited the lowest mean value (5.51 ± 1.02 mg/dL), while the highest mean LDL-C concentration was identified in group treated with STZ (8.70 ± 1.27 mg/dL). The absence of statistically significant differences was maintained across the treatment groups, including those receiving SGLT2 inhibitors either alone or together with Zn supplementation. These findings indicate that the experimental diabetes model and the administered interventions did not produce substantial changes in circulating LDL-C or HDL-C levels during the study period.

### 2.3. Histopathological and Immunohistochemical Evaluation of the Liver

**Figure 4:**
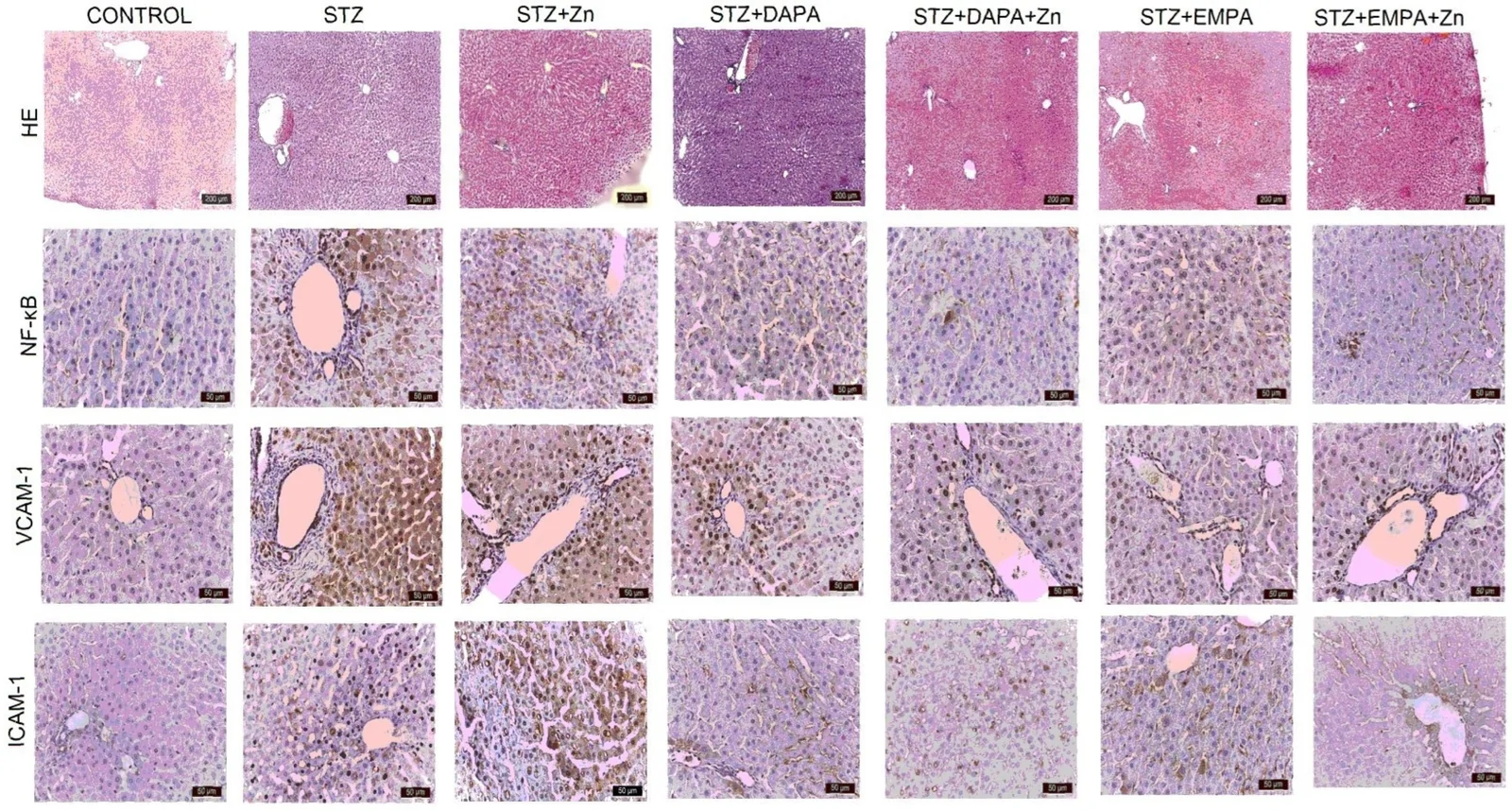
Representative liver histology from rats stained with Hematoxylin-Eosin (H&E, row 1) and immunohistochemically (IHC) stained for NF-κB p65 (row 2), VCAM-1 (row 3), and ICAM-1 (row 4), across the seven experimental groups: Control (untreated), STZ (streptozotocin-induced diabetes), STZ + Zn (STZ + zinc supplementation), STZ + DAPA (STZ + dapagliflozin), STZ + DAPA + Zn (STZ + dapagliflozin + zinc), STZ + EMPA (STZ + empagliflozin), and STZ + EMPA + Zn (STZ + empagliflozin + zinc). H&E staining shows overall hepatic architecture and hyperglycemia-induced changes; IHC staining shows the intensity and distribution of positive [brown/3,3′-diaminobenzidine DAB] immunostaining for NF-κB p65, VCAM-1, and ICAM-1, markers of hepatic inflammation and endothelial/leukocyte activation. Nuclei were counterstained with hematoxylin (blue). Scale bar = 200 μm (H&E), 50 μm (IHC).

Histological assessment of liver sections revealed well-preserved hepatic architecture in the Control group, with normal hepatocyte morphology, orderly hepatic cords, and intact sinusoidal spaces (Figure 4). In contrast, STZ group showed prominent structural alterations, including hepatocellular hydropic degeneration, steatosis, focal necrotic lesions, and sinusoidal dilatation, consistent with diabetes-associated hepatic injury. Treatment with DAPA or EMPA was associated with attenuation of these lesions, as evidenced by reduced hepatocellular degeneration and steatosis compared with the untreated diabetic group. Greater preservation of hepatic morphology was observed following the addition of Zn, particularly in the STZ + DAPA + Zn and STZ + EMPA + Zn groups. In these animals, only mild degenerative alterations were identified, and several microscopic fields exhibited an appearance closely resembling that of the Control group (Figure 4).

The histological findings were further supported by immunohistochemical assessment of hepatic NF-κB expression. Untreated diabetic rats displayed intense NF-κB immunoreactivity, consistent with enhanced activation of inflammatory signaling (Figure 4). Although marked immunoreactivity persisted in the STZ + Zn group, DAPA treatment resulted in a noticeable reduction in NF-κB expression, with an even greater decrease observed in the combination therapy groups. The lowest immunohistochemical signal was detected in the STZ + EMPA + Zn group, indicating the strongest attenuation of diabetes-associated inflammatory activity among the investigated treatment regimens (Figure 4).

A comparable immunohistochemical pattern was observed for the endothelial adhesion molecules VCAM-1 and ICAM-1 (Figure 4). In the untreated diabetic group, both markers showed strong immunoreactivity, predominantly localized to the sinusoidal endothelium, consistent with enhanced endothelial activation. Administration of SGLT2 inhibitors attenuated this response, with a further reduction in VCAM-1 and ICAM-1 staining following the addition of Zn. The weakest immunoreactivity was observed in the combined treatment groups, particularly in STZ + EMPA + Zn animals, where only minimal positive staining was detected (Figure 4). Collectively, these findings suggest that combined SGLT2 inhibition and Zn supplementation may limit diabetes-associated endothelial activation and leukocyte adhesion within the hepatic microvasculature.

Semiquantitative scoring corroborated these qualitative observations: overall differences among groups were significant (Kruskal–Wallis H = 31.47, df = 6, *p* < 0.001). The STZ group showed the highest hepatic injury score (steatosis, hepatocellular hypertrophy, and necrosis; median [IQR]: 10 [9-10]), which was significantly reduced following DAPA and EMPA treatment, with the lowest scores recorded in the STZ + EMPA + Zn group (3 [3-4]; *p* = 0.0054 vs. STZ, Dunn’s test with Bonferroni correction). Hepatic injury scores in the STZ + EMPA + Zn group did not differ significantly from the Control group (2 [2-2]; *p* = 1.000), indicating near-complete preservation of hepatic morphology in this group (Table 2).

Overall, the hepatic injury score showed a clear gradient of protection across treatment groups (Table 2). Only the STZ + EMPA + Zn group combined a significant reduction relative to STZ (*p* = 0.0054) with no significant difference from Control (*p* = 1.000), indicating that combined EMPA and Zn treatment most effectively normalized hepatic histology. The intermediate groups (STZ + DAPA, STZ + DAPA + Zn, and STZ + EMPA) showed descriptively lower scores than STZ, consistent with the qualitative improvement described above, but these differences did not reach statistical significance after Bonferroni correction for multiple comparisons–likely reflecting the conservative nature of this correction combined with the relatively small group size (n = 5), rather than an absence of a genuine treatment effect.

**Figure 5:**
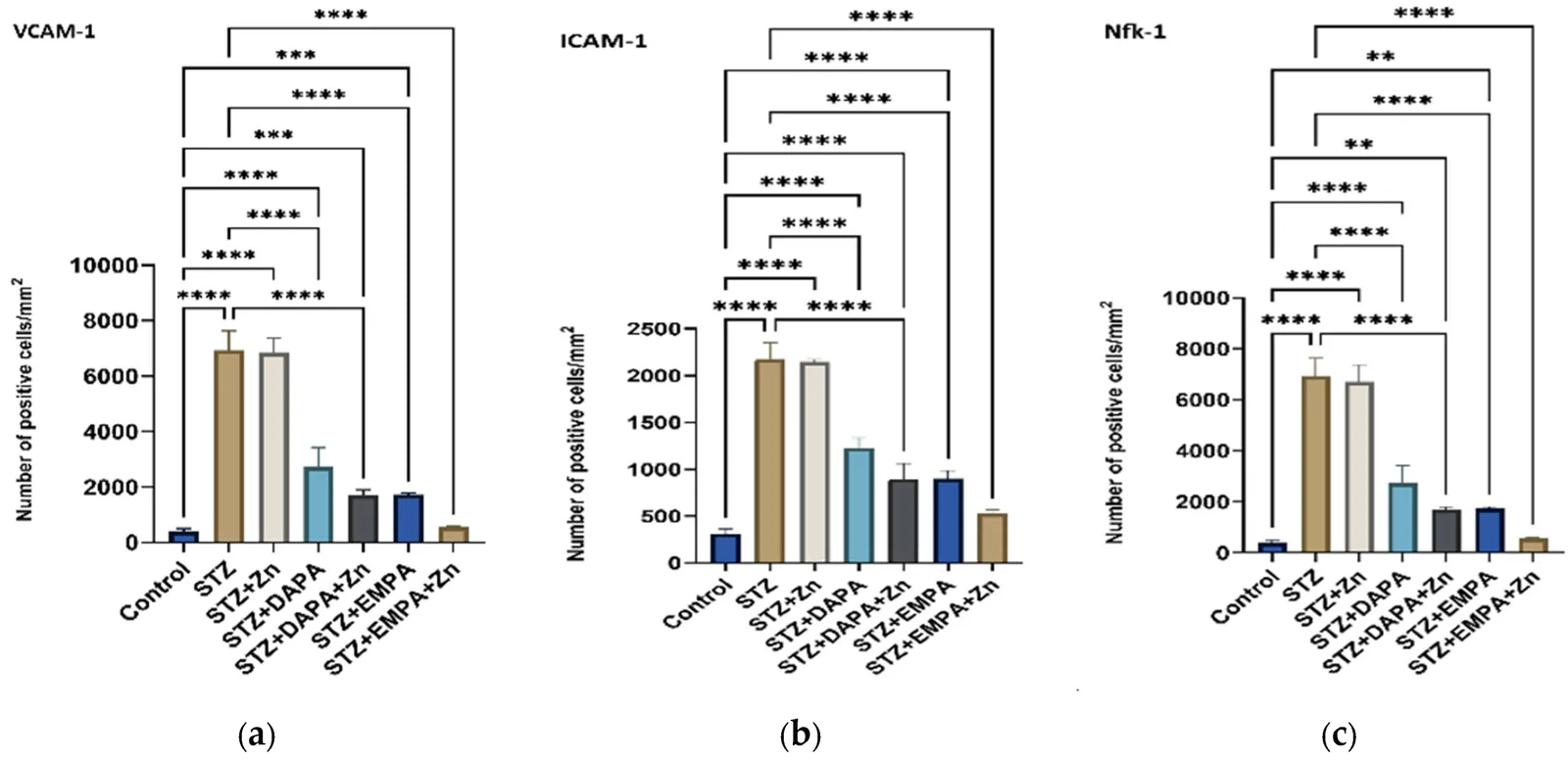
Immunohistochemical quantification of the number of positive cells/mm^2^ for (**a**). VCAM-1, (**b**). ICAM-1, (**c**). NF-κB p65, across the Control, STZ, STZ + Zn, STZ + DAPA, STZ + DAPA + Zn, STZ + EMPA, and STZ + EMPA + Zn groups. Data are expressed as mean ± SD. ** *p* < 0.01; *** *p* < 0.001; **** *p* < 0.0001.

Quantitative analysis further supported the immunohistochemical observations. STZ-induced diabetes resulted in a highly significant increase in VCAM-1- and ICAM-1-positive cells compared with the Control group (*p* < 0.0001). Zn supplementation alone did not significantly modify the expression of either adhesion molecule relative to untreated diabetic animals. In contrast, DAPA administration significantly decreased the number of VCAM-1- and ICAM-1-positive cells compared with the STZ group (*p* < 0.0001; Figure 5). Graphs were generated using GraphPad Prism 11.0.2, whereas statistical analyses, including one-way ANOVA followed by Tukey’s HSD post hoc test, were performed using IBM SPSS Statistics version 17.0.

The combination of DAPA and Zn (STZ + DAPA + Zn) resulted in a further significant reduction compared to STZ + DAPA alone (*p* < 0.0001), suggesting a greater improvement in the inflammatory profile. EMPA treatment (STZ + EMPA) also significantly decreased VCAM-1 and ICAM-1-positive cells relative to the STZ group. The combination of EMPA and Zn (STZ + EMPA + Zn) produced the lowest VCAM-1 (Figure 5a) and ICAM-1 (Figure 5b) expression levels, closely approximating those observed in the control group. The number of Nfk-1-positive cells was markedly increased in the STZ group compared to the control group (*p* < 0.0001). Zn administration alone (Stz + Zn) did not produce a significant effect. In contrast, groups treated with DAPA and EMPA (Stz + DAPA and Stz + EMPA) exhibited a significant reduction (*p* < 0.0001) in NFK-1-positive cells. Combinations of SGLT2 inhibitors and Zn (Stz + DAPA + Zn and Stz + EMPA + Zn) demonstrated improved efficacy, with the Stz + EMPA + Zn group showing results closely resembling those of the control group (Figure 5c).

### 2.4. Morphometric, Histopathological and Immunohistochemical Evaluation of the Pancreas

Morphometric analysis demonstrated significant diabetes-induced alterations in pancreatic morphology. Compared with the Control group, untreated diabetic rats exhibited a marked reduction in both the number of pancreatic islets and β-cell density. The mean number of islets decreased from 7.58 ± 0.80 islets/mm^2^ in the Control group to 2.40 ± 0.93 islets/mm^2^ in the STZ group, while the mean number of β-cells per islet declined from 85.4 ± 3.98 to 20.1 ± 0.93, indicating extensive destruction of the endocrine pancreas.

Zn supplementation alone produced only a modest improvement, increasing the number of pancreatic islets to 2.70 ± 0.76 islets/mm^2^ and the mean β-cell count to 29.2 ± 0.87 cells per islet. DAPA treatment partially restored pancreatic morphology, increasing the number of islets to 4.21 ± 0.48 islets/mm^2^ and the mean β-cell count to 43.2 ± 1.88 cells per islet. The addition of Zn was associated with more favorable changes in these parameters, with values reaching 5.63 ± 0.35 islets/mm^2^ and 51.1 ± 1.90 β-cells per islet. Among the therapeutic interventions, EMPA provided greater preservation of endocrine pancreatic morphology than DAPA, with mean values of 6.49 ± 0.58 islets/mm^2^ and 54.1 ± 2.98 β-cells per islet. The highest morphometric values among the treated groups were observed following combined EMPA and Zn administration (6.83 ± 0.67 islets/mm^2^ and 60.8 ± 1.98 β-cells per islet), approaching those recorded in non-diabetic animals.

**Figure 6:**
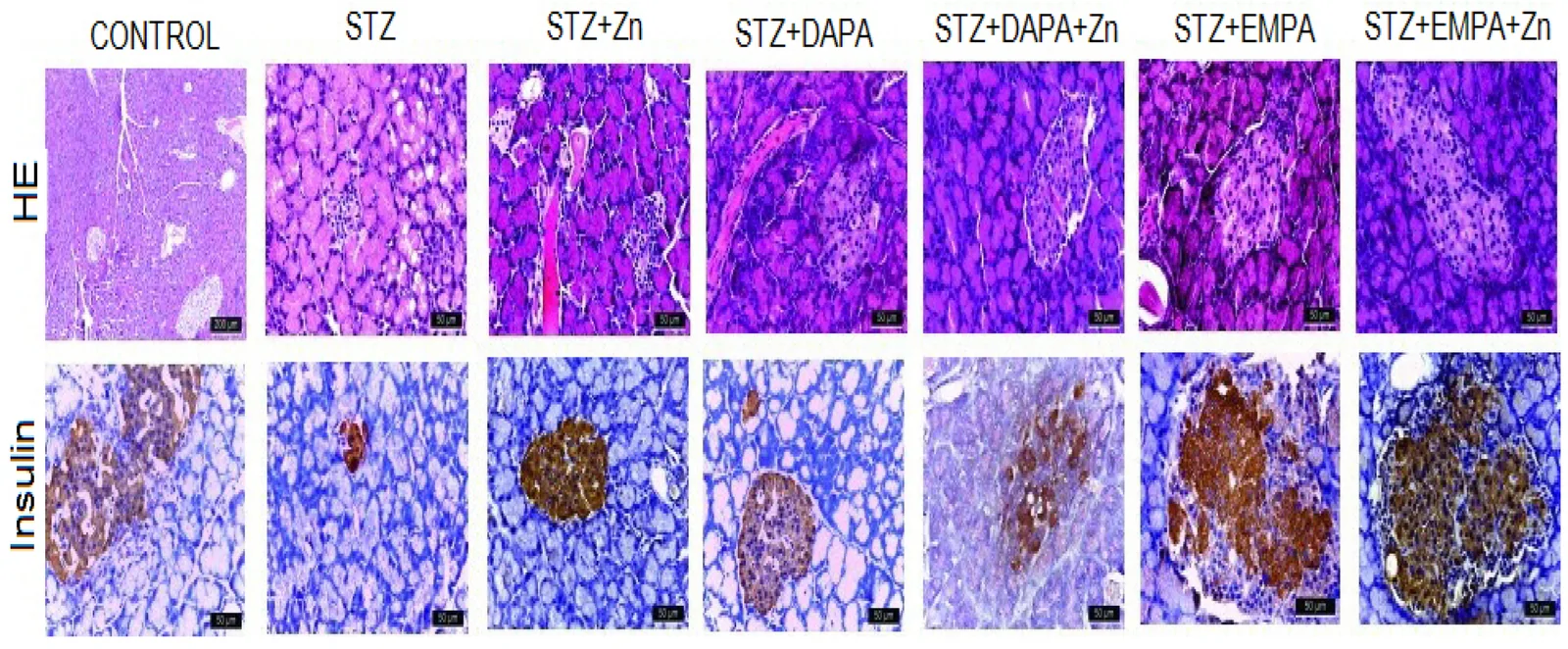
Representative H&E-stained pancreatic sections (row 1) illustrating the histopathological changes induced by diabetes and the protective effects of the investigated treatments, and immunohistochemical (IHC) staining with anti-insulin antibody (row 2), across the seven experimental groups: Control (untreated) (Barr 300 μm), STZ (STZ-induced diabetes), STZ + Zn (STZ + Zn supplementation), STZ + DAPA (STZ + DAPA), STZ + DAPA + Zn (STZ + DAPA + Zn), STZ + EMPA (STZ + EMPA), and STZ + EMPA + Zn (STZ + EMPA + Zn). H&E staining shows overall pancreatic architecture and STZ-induced changes in the islets of Langerhans; IHC staining shows the intensity and distribution of positive (brown/DAB) insulin immunostaining in pancreatic β-cells. Nuclei were counterstained with hematoxylin (blue). Barr 50 μm.

Histopathological assessment was consistent with the morphometric. Pancreatic tissue from the Control group maintained normal architecture, with clearly defined islets of Langerhans and preserved exocrine acini results (Figure 6).

In the STZ group, extensive structural damage was evident in both the endocrine and exocrine compartments, characterized by reduced islet size, pronounced β-cell depletion, nuclear pyknosis, and degenerative changes in the serous acini, together with intracellular lipid vacuolization. Zn supplementation alone resulted in only modest structural improvement. By comparison, treatment with either DAPA or EMPA was associated with attenuation of the diabetes-related lesions, including better preservation of islet morphology and reduced exocrine damage. The addition of Zn further strengthened these effects, with the STZ + EMPA + Zn group showing the closest resemblance to the pancreatic architecture of the Control animals and only minor residual alterations in the exocrine parenchyma.

Semiquantitative islet damage scoring corroborated the morphometric and histopathological findings: overall differences among groups were significant (Kruskal–Wallis H = 28.48, df = 6, *p* < 0.001). The STZ group exhibited the highest islet damage score (median [IQR]: 4 [4-4]), which decreased progressively with treatment, reaching the lowest values in the STZ + EMPA + Zn group (1 [1-2]; *p* = 0.024 vs. STZ, Dunn’s test with Bonferroni correction). Islet damage scores in the STZ + EMPA + Zn group did not differ significantly from the Control group (0 [0-1]; *p* = 1.000), consistent with near-complete preservation of islet architecture in this group (Table 3).

Islet damage scores followed a comparable pattern (Table 3). Neither the STZ + EMPA nor the STZ + EMPA + Zn group differed significantly from the Control group (*p* = 1.000 for both). However, only the STZ + EMPA + Zn group showed a statistically significant difference compared with the STZ group (*p* = 0.0238), indicating a significant improvement relative to the diabetic untreated group. DAPA-based regimens produced intermediate scores that did not differ significantly from either Control or STZ after correction, consistent with the comparatively moderate protective effect of DAPA observed throughout the study. Immunohistochemical analysis further demonstrated substantial changes in insulin-positive cell abundance following diabetes induction (Figure 6), indicating alterations in β-cell integrity and preservation. As circulating insulin and C-peptide concentrations were not assessed, these findings are interpreted as evidence of changes in β-cell insulin immunoreactivity rather than as a direct measure of pancreatic endocrine functional improvement. STZ administration markedly reduced the number of insulin-immunoreactive cells compared with the Control group (*p* < 0.0001), while Zn supplementation alone produced a profile comparable to that of untreated diabetic animals. Both DAPA and EMPA significantly increased the number of insulin-positive cells relative to the STZ group (*p* < 0.0001), with EMPA showing the more pronounced response. Importantly, co-administration of Zn further augmented the effects of both SGLT2 inhibitors, resulting in higher numbers of insulin-immunoreactive cells than those observed with DAPA or EMPA monotherapy (Figure 6).

**Figure 7:**
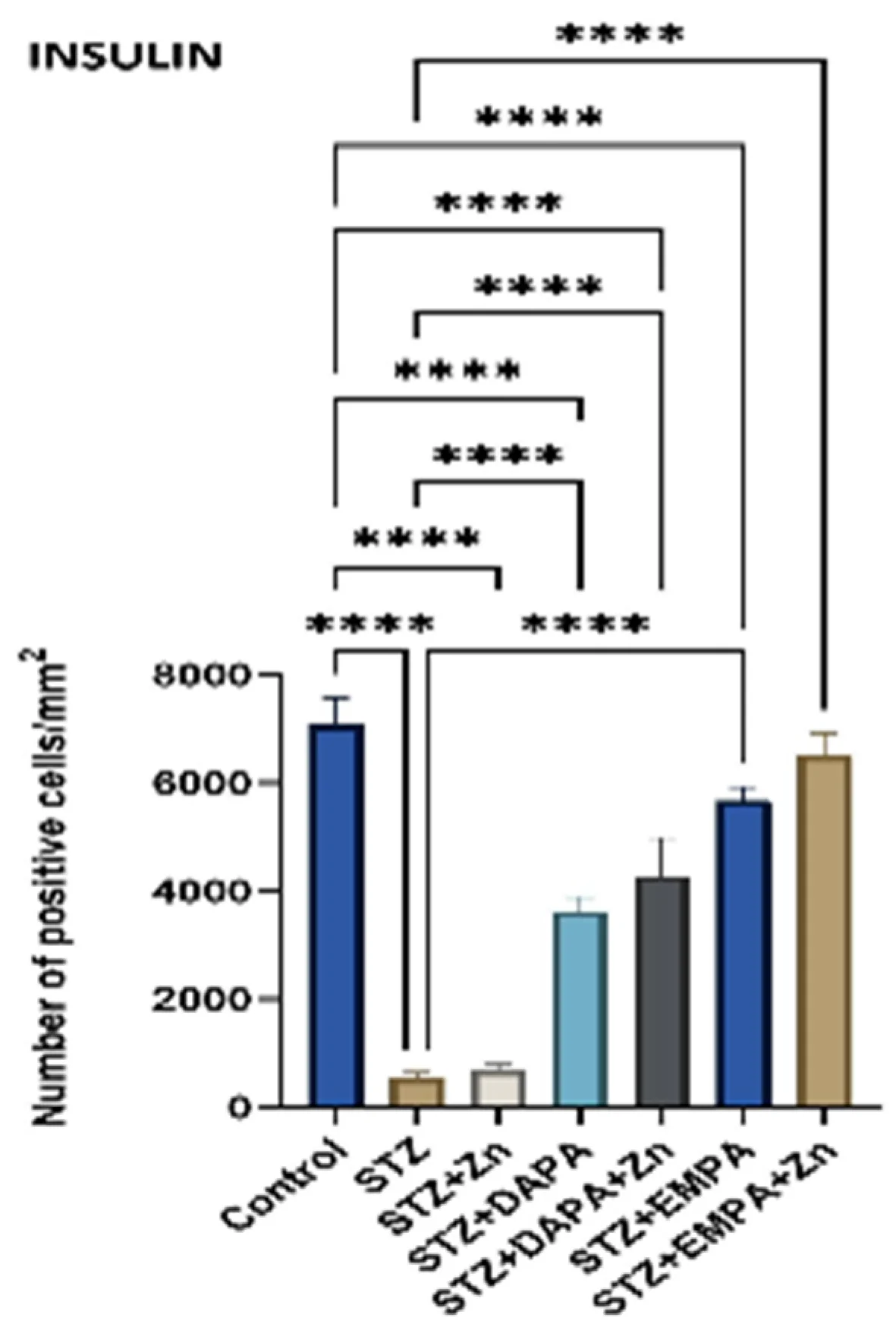
Immunohistochemical quantification of the number of positive cells/mm^2^ for insulin. In contrast to the inflammatory markers (Figure 5a–c), whose expression was significantly increased in the STZ group and progressively reduced by treatment, insulin expression was significantly decreased by STZ and progressively restored by the investigated treatments, with the STZ + EMPA + Zn group showing values closest to Control Data are expressed as mean ± SD. **** *p* < 0.0001.

Insulin immunohistochemistry provided further evidence of the treatment-related changes identified by morphometric and histopathological assessment (Figure 7). In the Control group, the islets of Langerhans exhibited strong and widespread insulin staining, consistent with preserved β-cell distribution. By contrast, STZ-induced diabetes was associated with a pronounced loss of insulin-positive cells, reflecting extensive β-cell depletion.

Zn supplementation alone resulted in only a modest increase in insulin immunoreactivity. A more evident preservation of insulin-positive β-cells was observed following treatment with DAPA or EMPA, suggesting partial restoration of the endocrine compartment. The most pronounced staining was detected after combined SGLT2 inhibitor and Zn administration, particularly in the STZ + EMPA + Zn group, where insulin-positive cells were abundantly distributed across both central and peripheral regions of the pancreatic islets. This pattern was consistent with the preservation of islet architecture and β-cell content observed in the morphometric and histopathological analyses, further supporting the potential beneficial effects of the EMPA + Zn combination.

## 3. Discussion

### 3.1. Findings in the Context of the Existing Literature

The confirmation of persistent hyperglycemia following STZ administration validated the establishment of the experimental diabetic model and provided the metabolic context necessary for interpreting the hepatic and pancreatic alterations observed in the present study. STZ-induced diabetes represents a widely used experimental model of insulin deficiency that reproduces several metabolic disturbances associated with chronic hyperglycemia, including impaired glucose homeostasis, inflammation, cellular injury, and progressive tissue dysfunction [60,70,71]. Although detailed glycemic results have been previously reported, the improvement in blood glucose levels observed following DAPA or EMPA treatment is relevant for understanding the protective effects identified in hepatic and pancreatic tissues. Reduction in chronic hyperglycemia limits glucotoxicity and decreases the activation of pathways involved in diabetes-associated cellular injury, thereby contributing to preservation of tissue structure and function [40,72].

Importantly, the hepatic and pancreatic findings observed in the present study should be interpreted in the context of the metabolic improvements previously documented in the same experimental cohort [60]. The reduction in fasting blood glucose following SGLT2 inhibitor treatment provides a plausible explanation for at least part of the observed tissue protection, as attenuation of chronic hyperglycemia may reduce glucotoxic stress and its downstream inflammatory and oxidative consequences. Nevertheless, the concomitant reduction in hepatic NF-κB, VCAM-1, and ICAM-1 expression, together with the improvement in hepatic architecture and preservation of pancreatic β-cell morphology, suggests that mechanisms in addition to improved glycemic control may contribute to the observed effects. Because the present study was not designed to distinguish glycemia-mediated from glycemia-independent effects and no formal mediation analysis was performed, the hepatic and pancreatic protective effects should not be interpreted as being definitively independent of glycemic control. Rather, improved metabolic control and attenuation of inflammatory and tissue-injury pathways may represent complementary mechanisms underlying the observed protection. Increasing evidence indicates that these agents exert pleiotropic actions, including modulation of inflammatory pathways, improvement of cellular metabolism, and protection against diabetes-associated organ damage [73,74]. Therefore, the hepatic and pancreatic improvements observed in the present study likely result from a combination of improved metabolic control and attenuation of pathogenic mechanisms involved in diabetic tissue injury.

The biochemical findings demonstrated clear evidence of hepatic involvement in untreated diabetic animals. Increased serum ALT, AST, LDH, and ALP activities in the STZ group indicate hepatocellular injury, altered membrane integrity, and impaired hepatic function. These changes are consistent with previous reports showing that chronic hyperglycemia promotes metabolic overload, inflammatory activation, and structural damage in the liver [75,76].

ALT is considered a sensitive indicator of hepatocellular injury due to its predominant localization within hepatocytes [77]. The marked increase in ALT activity observed in untreated diabetic rats was consistent with the histological findings of hepatocyte degeneration, steatosis, and focal necrotic changes. Administration of DAPA or EMPA reduced ALT levels compared with untreated diabetic animals, suggesting improved preservation of hepatocyte integrity. Nevertheless, enzyme activity remained above control values, indicating that treatment attenuated, but did not completely reverse, diabetes-induced hepatic injury during the experimental period. Similar reductions in hepatic enzymes following SGLT2 inhibitor therapy have been reported in experimental T2DM and in patients with metabolic dysfunction-associated steatotic liver disease [45,78].

AST showed a comparable pattern, with the highest activity detected in untreated diabetic rats and reduced values following treatment. Because AST is present in both cytosolic and mitochondrial compartments of hepatocytes, as well as in extrahepatic tissues, its elevation reflects a broader pattern of tissue injury associated with diabetes [79]. The partial reduction observed after SGLT2 inhibitor administration suggests a protective effect against diabetes-associated cellular damage, although the persistence of mildly increased values indicates incomplete restoration of normal tissue homeostasis.

LDH exhibited a pronounced increase following diabetes induction, reflecting increased cellular injury and membrane permeability [80]. Unlike ALT and AST, LDH is distributed across multiple tissues; therefore, its elevation may indicate generalized metabolic injury associated with severe hyperglycemia. The reduction in LDH activity following treatment supports the protective effect of SGLT2 inhibition on tissue integrity, although the incomplete normalization suggests the persistence of residual metabolic alterations.

Increased ALP activity in diabetic animals further indicated impairment of hepatic function. Elevated ALP levels have been associated with diabetes-related hepatic alterations and may reflect disturbances in hepatobiliary function, membrane turnover, and inflammatory processes [81]. Although DAPA and EMPA reduced ALP activity compared with untreated diabetic rats, values remained higher than those observed in controls, in agreement with the residual histological alterations identified in treated animals.

The biochemical alterations observed in diabetic animals were supported by the histopathological findings, demonstrating that diabetes-induced hepatic injury was associated with structural deterioration of the liver. Untreated diabetic rats exhibited hepatocellular degeneration, steatosis, focal necrotic changes, and sinusoidal alterations, indicating that chronic metabolic stress affected both hepatocyte integrity and hepatic architecture. In contrast, treatment with DAPA or EMPA reduced the severity of these alterations, while the addition of Zn resulted in a more pronounced preservation of hepatic structure. The improved morphology observed in the combination therapy groups was consistent with the lower circulating levels of hepatic enzymes, suggesting that the biochemical improvements reflected reduced tissue injury rather than isolated changes in serum parameters.

The reduction in hepatic NF-κB immunoreactivity following treatment provides additional evidence for attenuation of diabetes-associated inflammatory activation. NF-κB represents a central regulator of inflammatory responses and is activated by several metabolic stressors associated with diabetes, including hyperglycemia, advanced glycation end products, free fatty acids, and inflammatory mediators. Activation of this pathway promotes the expression of pro-inflammatory cytokines and contributes to hepatocellular injury and tissue remodeling [32,82]. Therefore, the increased NF-κB expression observed in untreated diabetic animals is consistent with persistent inflammatory activation, whereas the reduced immunoreactivity detected after SGLT2 inhibitor administration suggests suppression of inflammatory signaling.

A similar pattern was observed for the endothelial adhesion molecules VCAM-1 and ICAM-1. Increased expression of these markers in the STZ group indicates endothelial activation and enhanced inflammatory cell recruitment, processes that contribute to the progression of diabetes-associated tissue injury. The reduction in VCAM-1 and ICAM-1 immunostaining following treatment suggests that DAPA and EMPA may attenuate vascular inflammatory responses within the hepatic microenvironment. These findings are consistent with previous evidence describing anti-inflammatory and vascular protective effects associated with SGLT2 inhibition [83].

The combination therapy groups, particularly STZ + EMPA + Zn, exhibited the most favorable histological and immunohistochemical profile, suggesting a greaterprotective effect when Zn was added to SGLT2 inhibitor treatment. Zn may contribute to tissue protection through several complementary mechanisms, including regulation of cellular metabolism, stabilization of cellular membranes, modulation of inflammatory signaling, and support of endogenous antioxidant systems. Although oxidative stress markers were not directly evaluated in the present study, previous experimental evidence indicates that Zn availability influences redox homeostasis and inflammatory responses in diabetic conditions [54,59,84]. Therefore, the beneficial effects observed after Zn supplementation should be interpreted as consistent with, rather than direct evidence of, improved antioxidant defense.

The improved response observed with combined therapy indicates that the addition of Zn was associated with greater treatment-related improvements for several of the investigated endpoints. These observations are consistent with the possibility that SGLT2 inhibition and Zn supplementation may provide complementary biological effects through partially overlapping or distinct mechanisms. However, because no formal Drug × Zn interaction analysis was performed, the present findings cannot establish a statistical interaction, additivity, or pharmacological synergy between the two interventions. The observed differences should therefore be interpreted as treatment-associated complementary effects that warrant confirmation in specifically designed factorial studies. While SGLT2 inhibitors primarily improve glucose handling through enhanced urinary glucose excretion, their additional effects on inflammatory pathways and tissue protection may contribute to organ preservation. Zn supplementation may further support these effects by improving cellular resilience under diabetic conditions. Consequently, the favorable hepatic outcomes observed in the combination groups indicate that targeting multiple interconnected pathways may provide broader benefits than glucose lowering alone.

The present findings also highlight the importance of interpreting hepatic protection as an integrated response involving biochemical, structural, and inflammatory parameters. The concordance between reduced serum enzyme activities, improved hepatic morphology, and decreased NF-κB, VCAM-1, and ICAM-1 expression provides consistent evidence that DAPA and EMPA exert protective effects at both functional and structural levels. Importantly, these improvements were most evident when SGLT2 inhibitors were combined with Zn supplementation, supporting the potential value of adjunctive strategies in limiting diabetes-associated hepatic injury.

The pancreatic findings provide important evidence that the beneficial effects of SGLT2 inhibition extend beyond systemic metabolic improvement and involve preservation of endocrine pancreatic structure. STZ administration produced profound alterations in pancreatic morphology, characterized by a marked reduction in the number of islets of Langerhans, decreased β-cell density, and extensive loss of insulin-immunoreactive cells. These findings are consistent with the well-established susceptibility of pancreatic β-cells to diabetic injury, as these cells possess relatively limited intrinsic antioxidant defense capacity and are therefore highly vulnerable to metabolic stress associated with chronic hyperglycemia [85].

The reduction in β-cell mass observed in untreated diabetic animals represents a central feature of STZ-induced diabetes and results from direct pancreatic toxicity combined with the consequences of persistent hyperglycemia. Loss of functional β-cell population leads to impaired insulin production and further aggravates metabolic disturbances, creating a vicious cycle of hyperglycemia and tissue injury [86,87]. In the present study, the severe reduction in islet number and β-cell density in the STZ group was accompanied by marked histopathological alterations, confirming extensive endocrine pancreatic damage.

Treatment with DAPA or EMPA partially preserved pancreatic architecture, as demonstrated by increased islet number, higher β-cell density, and greater insulin immunoreactivity compared with untreated diabetic animals. These findings suggest that improvement of glycemic control through SGLT2 inhibition was associated with preservation of pancreatic endocrine tissue. Although the mechanisms responsible for this effect were not directly investigated, previous studies indicate that SGLT2 inhibitors may exert pancreatic protective actions through reduction in glucotoxicity, improvement of metabolic flexibility, and modulation of inflammatory pathways involved in β-cell dysfunction [48,86].

A particularly important finding of the present study was the greater improvement in pancreatic outcomes observed following the combined administration of SGLT2 inhibitors and Zn supplementation. The STZ + EMPA + Zn group exhibited the highest number of pancreatic islets, the greatest β-cell density, and the strongest insulin immunoreactivity among treated animals, approaching values observed in non-diabetic controls. These findings indicate that Zn supplementation potentiated the beneficial effects of SGLT2 inhibition on pancreatic endocrine preservation.

The potential contribution of Zn to β-cell protection is biologically plausible. Zn is essential for insulin biosynthesis, storage, and crystallization within pancreatic β-cells and participates in the regulation of insulin secretion. In addition, Zn influences cellular defense mechanisms and inflammatory responses, which may contribute to maintaining β-cell viability under diabetic conditions [52,88]. Although these mechanisms were not directly measured in the present study, the improved morphometric and immunohistochemical outcomes observed after Zn supplementation are consistent with previous evidence supporting the importance of Zn availability for pancreatic function [89,90].

The differential response observed with EMPA-based combination therapy may reflect differences in pharmacological activity between individual SGLT2 inhibitors, although the precise mechanisms underlying this observation require further investigation. The stronger preservation of pancreatic morphology in the STZ + EMPA + Zn group was accompanied by improved hepatic parameters, suggesting that protection of endocrine pancreatic tissue occurred in parallel with improvement of systemic metabolic status. This coordinated response highlights the importance of considering diabetes-associated organ injury as a network of interconnected alterations rather than as isolated dysfunction of individual organs.

The simultaneous improvement of hepatic and pancreatic parameters supports the concept of a liver–pancreas metabolic axis in diabetes. Chronic hyperglycemia, altered lipid metabolism, inflammatory activation, and metabolic overload contribute to dysfunction of both organs through interconnected pathways. Pancreatic β-cell impairment promotes hepatic metabolic disturbances through reduced insulin availability, while hepatic lipid accumulation and inflammatory signaling further contribute to systemic metabolic deterioration [9,10]. Therefore, therapeutic strategies capable of improving both glucose handling and tissue homeostasis may provide broader benefits than interventions focused exclusively on glycemic reduction.

In this context, the present findings suggest that SGLT2 inhibition combined with Zn supplementation acts through complementary mechanisms that preserve both hepatic and pancreatic integrity. The agreement between pancreatic morphometric analysis, insulin immunostaining, liver enzyme profiles, hepatic histology, and inflammatory marker expression provides integrated evidence supporting a multi-organ protective effect. These results emphasize the potential value of combination strategies aimed at preserving metabolic organ function in diabetes-associated complications.

Taken together, the present findings extend the evaluation of SGLT2 inhibitor therapy beyond glycemic control by demonstrating concordant changes across biochemical, structural, inflammatory, and pancreatic tissue parameters in an experimental model of diabetes. The relevance of these findings lies in the integrated assessment of the liver–pancreas axis, which may provide a broader framework for understanding diabetes-associated organ injury and the potential effects of adjunctive nutritional interventions. The more pronounced tissue preservation observed with the combined SGLT2 inhibitor–Zn regimens, particularly EMPA + Zn, provides an experimental rationale for further investigation of combined metabolic and nutritional approaches. However, these findings should be regarded as hypothesis-generating rather than as evidence of clinical efficacy, as the present experimental design does not establish the mechanisms of combination therapy or its clinical applicability.

### 3.2. Study Limitations

Several limitations of the present study should be acknowledged when interpreting the findings. First, the experimental model was based on STZ-induced diabetes, which primarily reflects pancreatic β-cell injury and insulin deficiency rather than the complex pathophysiology of human T2DM, characterized by insulin resistance, obesity, chronic low-grade inflammation, and metabolic syndrome. Therefore, translation of these findings to patients with T2DM or metabolic dysfunction-associated steatotic liver disease should be approached with caution.

Second, the duration of the intervention may have limited the evaluation of long-term effects of DAPA, EMPA and Zn supplementation on progressive hepatic remodeling, fibrosis development, and sustained metabolic adaptation. Although significant improvements were observed in biochemical parameters, tissue architecture, and inflammatory markers, longer treatment periods will be necessary to determine whether these interventions can prevent or reverse advanced metabolic complications.

Another limitation is the relatively small number of animals included in each experimental group, which may have reduced the statistical power for detecting moderate treatment effects in certain parameters. This aspect may partially explain the absence of significant differences in some metabolic variables, including serum lipid parameters, despite observable trends toward improvement. Future studies with larger cohorts may provide more precise estimates of treatment effects.

The present investigation evaluated biochemical alterations together with histopathological, morphometric, and immunohistochemical changes. Although the reduced expression of NF-κB, VCAM-1, and ICAM-1 suggests attenuation of inflammatory and endothelial activation pathways, the molecular mechanisms responsible for these effects were not directly investigated. Additional molecular analyses targeting inflammatory, oxidative, mitochondrial, and lipid metabolism pathways would further clarify the mechanisms underlying the protective actions of SGLT2 inhibitors and Zn supplementation.

Furthermore, direct measurements of hepatic lipid accumulation and lipid metabolism regulation were not performed. This limitation is relevant because circulating lipid parameters showed relatively modest changes, whereas histological examination demonstrated clear differences in hepatic steatosis between experimental groups. Future studies incorporating hepatic lipid quantification and evaluation of key regulators of lipid metabolism may provide additional insight into the mechanisms responsible for the observed morphological improvement.

Although combination therapy produced the most favorable outcomes, potential pharmacokinetic or molecular interactions between SGLT2 inhibitors and Zn supplementation were not evaluated. Further investigations are required to clarify the nature and mechanisms underlying the additional effects observed with Zn supplementation in combination with SGLT2 inhibitors.

While pancreatic structural preservation was demonstrated through morphometric analysis and insulin immunostaining, functional assessment of pancreatic recovery was not performed. Future studies incorporating dynamic measurements of insulin secretion and β-cell function would provide a more comprehensive understanding of the effects of these therapeutic strategies.

Although the observed preservation of pancreatic islet density and β-cell insulin immunoreactivity suggests a protective effect on pancreatic islet integrity, these findings should not be interpreted as direct evidence of improved endocrine function. A limitation of the present study is the absence of circulating functional biomarkers such as fasting insulin and C-peptide, as well as derived indices such as HOMA-IR and HOMA-β. Therefore, the functional significance of the observed morphological and immunohistochemical changes remains to be established. Future studies incorporating direct assessments of endocrine pancreatic function will be necessary to determine whether the observed preservation of β-cell integrity translates into improved insulin secretory capacity and metabolic function.

Other limitation of the present study is that the histological scoring and morphometric assessments were not performed under blinded conditions. Although the animals were randomly allocated to the experimental groups, the evaluator was aware of the group allocation during the assessment. Consequently, the possibility of observer-related bias cannot be completely excluded. Future studies should incorporate blinded assessment of histological and morphometric endpoints to further minimize this potential source of bias.

The histopathological assessment of pancreatic islet damage was based on a scoring system developed specifically for the present study, which has not undergone independent validation against an established or externally validated scoring system. Therefore, the findings derived from this assessment should be considered exploratory and interpreted with appropriate caution. Future studies employing independently validated or standardized histopathological scoring systems are warranted to confirm these observations. Despite these limitations, the present study provides complementary biochemical, structural, and immunohistochemical findings that support the potential protective effects of DAPA, EMPA, and Zn supplementation against diabetes-associated hepatic and pancreatic alterations. Further studies using complementary experimental models, longer treatment periods, and expanded molecular analyses are required to confirm these findings and clarify their translational relevance.

### 3.3. Future and Translational Implications

The findings of the present study provide experimental evidence supporting the potential of SGLT2 inhibitors, particularly when combined with Zn supplementation, as a strategy for attenuating diabetes-associated hepatic and pancreatic injury. The observed improvements in liver morphology, reduction in circulating markers of tissue damage, preservation of pancreatic β-cell structure, and attenuation of inflammatory pathways suggest that these interventions may provide benefits beyond glucose regulation alone. Considering the increasing recognition of metabolic dysfunction-associated steatotic liver disease (MASLD) as an important complication of diabetes, therapeutic approaches capable of improving metabolic control while limiting tissue inflammation and injury represent an important area of investigation.

From a translational perspective, the hepatoprotective effects observed with DAPA and EMPA may be relevant for patients with T2DM who are at increased risk of hepatic steatosis and progressive liver dysfunction. Although SGLT2 inhibitors are primarily prescribed for glycemic control and cardiovascular or renal protection, accumulating clinical evidence suggests additional beneficial effects on hepatic steatosis, liver enzyme profiles, and inflammatory status. The present findings further support the concept that these effects may involve modulation of inflammatory signaling pathways, including NF-κB activation and endothelial responses.

The potential complementary interaction between SGLT2 inhibitors and Zn supplementation represents an additional area of translational interest. Zn is an accessible micronutrient involved in insulin metabolism, cellular protection, immune regulation, and maintenance of tissue integrity. The histological and immunohistochemical findings observed in animals receiving combined therapy suggest that Zn supplementation may contribute to the observed effects of SGLT2 inhibition, potentially through complementary biological mechanisms. However, clinical application requires careful evaluation of optimal dosage, treatment duration, baseline Zn status, and potential interactions with existing therapies.

Future experimental studies should investigate these findings in models that more closely reproduce human metabolic disease, including diet-induced obesity, insulin resistance, and MASLD models. Longer intervention periods will be necessary to determine whether combined SGLT2 inhibitor and Zn therapy can influence progression from hepatic steatosis toward more advanced stages of liver disease. Additional molecular studies evaluating inflammatory regulation, lipid metabolism, mitochondrial function, and cellular stress responses may further clarify the mechanisms underlying these protective effects.

From a clinical research perspective, prospective studies are required to determine whether Zn supplementation provides additional hepatic benefits in patients already receiving SGLT2 inhibitors. Such investigations should include assessment of liver enzymes, non-invasive fibrosis markers, imaging-based evaluation of hepatic fat accumulation, inflammatory parameters, and metabolic outcomes. Identification of patient populations most likely to benefit from combined metabolic and nutritional interventions may contribute to more individualized therapeutic strategies.

The present study supports the concept that diabetes-associated organ complications may require therapeutic approaches targeting multiple interconnected mechanisms. Overall, the present findings provide experimental support for further investigation of combined SGLT2 inhibition and Zn supplementation as a potential approach to diabetes-associated hepatic and pancreatic injury. The observed concordance between biochemical, histopathological, morphometric, and immunohistochemical outcomes supports the relevance of an integrated liver–pancreas perspective. Nevertheless, the translational implications remain preliminary because the present study was conducted in a small, male-only experimental cohort, over a relatively short treatment period, and did not include direct assessments of pancreatic endocrine function, hepatic lipid metabolism, or the molecular mechanisms underlying the observed effects. Future studies using metabolically relevant animal models, larger cohorts, longer interventions, and direct functional and molecular assessments are therefore required before the potential clinical relevance of combined SGLT2 inhibitor and Zn supplementation can be established.

## 4. Materials and Methods

### 4.1. Substances

DAPA and EMPA were administered using their commercially available tablet formulations to reproduce clinically relevant treatment conditions. DAPA was administered in a 10 mg tablet formulation, whereas EMPA was administered in a 25 mg tablet formulation. The tablets were prepared for oral administration according to the experimental dosing protocol. STZ (~75% β-anomer, ≥98% purity by HPLC, molecular weight 265.22, catalogue no. S0130), citrate buffer (0.09 M; catalogue no. C2488), Zn chloride (molecular weight 136.3, catalogue no. 208086), and distilled water (molecular weight 18.02, catalogue no. 07-6061) were purchased from Sigma-Aldrich (Steinheim, Germany; www.sigma-aldrich.com; accessed 26 June 2026).

### 4.2. Animals

Thirty-five male Sprague–Dawley rats, aged 8–10 weeks and weighing between 200 and 250 g, wererandomly assigned to seven experimental groups (n = 5 rats/group): Control, STZ, STZ + Zn, STZ + DAPA, STZ + DAPA + Zn, STZ + EMPA, and STZ + EMPA + Zn. Random allocation was performed prior to the initiation of the treatment protocol. The animals were obtained from the Cantacuzino National Medical-Military Research and Development Institute (Băneasa, Bucharest, Romania) and maintained under standardized laboratory conditions, including a controlled ambient temperature of 22 ± 2 °C, a 12 h light/12 h dark cycle, unrestricted access to drinking water, and standard housing throughout the study. Male rats were exclusively selected in the present study to maintain a homogeneous experimental population, as sex-related differences may influence susceptibility to experimental diabetes and the response to STZ-induced pancreatic β-cell injury. Previous evidence indicates that sex hormones, particularly estrogens, may exert protective effects on pancreatic β-cell survival and function and may consequently contribute to sex-related differences in diabetes susceptibility [91]. Therefore, the use of male animals was considered appropriate for establishing a relatively homogeneous experimental model; however, this design does not permit direct evaluation of sex-dependent differences in the effects of the investigated treatments. The Sprague–Dawley strain was selected because it is a well-established experimental model extensively used in metabolic and diabetes research [92].

### 4.3. Ethical Aspects

The experimental protocol was reviewed and approved by the Institutional Ethics Committee of the Grigore T. Popa University of Medicine and Pharmacy, Iași (Approval No. 30/14 January 2021), and by the Veterinary Public Health Directorate (Authorization No. 30/24 February 2021). All experimental procedures complied with the applicable national regulations and internationally accepted guidelines governing the care and use of laboratory animals. Every effort was made to reduce animal discomfort and to adhere to the principles of humane experimental practice throughout the study [93,94].

### 4.4. Experimental Protocol

T2DM-like metabolic alterations were induced using a combined HFD and low-dose STZ protocol. This experimental model was selected to reproduce selected metabolic and pancreatic features relevant to diabetes, including hyperglycemia, insulin resistance, and β-cell injury, while providing an established experimental framework for investigating diabetes-associated hepatic and pancreatic alterations. Given the limitations inherent to experimental models, the findings obtained using this approach should be interpreted with caution when extrapolating to the heterogeneous pathophysiology of human T2DM [95].

All experimental procedures were performed at the CEMEX–”Ostin C. Mungiu” Advanced Research and Development Center for Experimental Medicine, Grigore T. Popa University of Medicine and Pharmacy, Iași, Romania. Throughout the study, animals were maintained under standardized environmental conditions, and all experimental procedures were carried out according to the same protocol.

#### 4.4.1. Induction of Experimental T2DM

To establish a diet-associated metabolic background prior to diabetes induction, rats assigned to the diabetic groups were initially fed a HFD for two consecutive weeks. The diet, developed in collaboration with the Cantacuzino National Medical-Military Research and Development Institute (Băneasa, Bucharest, Romania), consisted of 45% fat, 40% carbohydrates, and 15% protein and was administered at 25 g/animal/day. The HFD contained corn, wheat, barley, soybean meal, sunflower meal, full-fat soy, lard, sugar, monocalcium phosphate, methionine, calcium carbonate, sodium chloride, and a vitamin-mineral premix formulated for laboratory rodents.

Following the 2-week HFD period, animals were fasted for 12 h and fasting blood glucose was measured. Diabetes was subsequently induced by a single intraperitoneal injection of STZ (35 mg/kg body weight), freshly dissolved in 0.1 M citrate buffer (pH 4.5). The freshly prepared STZ solution was administered promptly after preparation to minimize degradation. Control animals received the corresponding citrate-buffer vehicle.

Fasting blood glucose was measured 72 h after STZ administration, and animals with glucose concentrations >250 mg/dL were considered diabetic. Fasting blood glucose was reassessed 7 days after STZ administration to confirm the persistence of the hyperglycemic state before initiation of the treatment phase.

Following confirmation of diabetes, the experimental treatments were administered once daily by oral gavage for four weeks. DAPA (1 mg/kg/day), EMPA (10 mg/kg/day), and Zn chloride (5 mg/kg/day) were administered once daily by oral gavage for four consecutive weeks according to the experimental design. The seven experimental groups were: Control, STZ, STZ + Zn, STZ + DAPA, STZ + DAPA + Zn, STZ + EMPA, and STZ + EMPA +Zn (n = 5/group). The STZ + Zn group received ZnCl_2_ alone (5 mg/kg/day), whereas the STZ + DAPA + Zn and STZ + EMPA + Zn groups received the respective SGLT2 inhibitor in combination with ZnCl_2_ at the doses indicated above. Thus, the combination treatments consisted of DAPA + ZnCl_2_ (1 + 5 mg/kg/day) or EMPA + ZnCl_2_ (10 + 5 mg/kg/day). The STZ group served as the untreated diabetic group, receiving no pharmacological intervention, while non-diabetic control animals received the citrate-buffer vehicle. Fasting blood glucose was reassessed at the end of the treatment period to evaluate the metabolic response to the respective interventions. The HFD/STZ induction protocol and treatment doses were based on our previously published experimental methodology [60] and are described here in detail, with the text fully rephrased, to ensure methodological transparency and reproducibility.

#### 4.4.2. Blood Collection and Biochemical Assessment

Blood samples (0.3 mL) were collected from the lateral tail vein at two predefined experimental time points: before dietary intervention and at the end of the four-week treatment period. Blood glucose concentrations were assessed after a 12 h fasting period at baseline and at the end of the four-week treatment period, as previously described [60]. Animals with fasting blood glucose concentrations >250 mg/dL following STZ administration were considered diabetic. The present study subsequently focused on biochemical, histopathological, morphometric, and immunohistochemical parameters related to hepatic and pancreatic injury. Biochemical assessment included serum markers of hepatic injury and lipid metabolism. Hepatic injury was evaluated by measuring ALT, AST, LDH, and ALP. Lipid metabolism was assessed by measuring total cholesterol and TG concentrations.

The biochemical analyses were performed using an automated biochemical analyzer based on spectrophotometric measurements. Serum was separated by centrifugation and subsequently analyzed at Praxis Laboratory (Iași, Romania). The determination of ALT, AST, LDH, and ALP enzymes was performed using biochemical spectrophotometric methods, mainly by measuring enzymatic activity kinetically. For ALT, the reaction involved the conversion of alanine and α-ketoglutarate into pyruvate and glutamate, and the enzyme activity was determined indirectly by measuring NADH (nicotinamide adenine dinucleotide, reduced form) consumption at 340 nm. For AST, aspartate and α-ketoglutarate were converted into oxaloacetate and glutamate, and oxaloacetate was subsequently converted into malate, with NADH consumption; the decrease in absorbance at 340 nm was proportional to AST activity. LDH catalyzed the reversible conversion of pyruvate into lactate, accompanied by the oxidation of NADH to NAD^+^ (nicotinamide adenine dinucleotide, oxidized form), and enzyme activity was determined by monitoring the change in NADH absorbance at 340 nm. In the case of ALP, the enzyme hydrolyzed p-nitrophenyl phosphate in an alkaline medium, producing p-nitrophenol, and the reaction product was measured spectrophotometrically at 405 nm.

The lipid profile was assessed by biochemical techniques, primarily based on enzymatic and spectrophotometric methods. Total cholesterol was determined using the enzymatic colorimetric CHOD-PAP (Cholesterol Oxidase–Peroxidase–Aminophenazone) method, in which cholesterol was oxidized and the hydrogen peroxide produced participated in a chromogenic reaction; the intensity of the color was proportional to the cholesterol concentration. TG were determined using the enzymatic colorimetric GPO-PAP (Glycerol Phosphate Oxidase–Peroxidase–Aminophenazone) method, which involved the hydrolysis of TG and the conversion of glycerol into products that led to the formation of hydrogen peroxide and, subsequently, a colored compound that was measured spectrophotometrically. HDL-C and LDL-C were determined directly using homogeneous enzymatic colorimetric methods, with specific reagents that selectively allowed the measurement of cholesterol contained in HDL and LDL particles, respectively.

#### 4.4.3. Statistical Analysis

Continuous variables are presented as mean ± standard deviation (SD). Statistical analyses were performed using IBM SPSS Statistics software (version 17.0; IBM Corp., Armonk, NY, USA). Differences among the experimental groups were evaluated using one-way analysis of variance (ANOVA), followed by Tukey’s HSD post hoc test for multiple comparisons. Statistical significance was established at *p* < 0.05.

For variables analyzed using a non-parametric approach, differences among groups were evaluated using the Kruskal–Wallis test, followed by Dunn’s post hoc test with Bonferroni correction for multiple comparisons.

For pairwise comparisons, standardized effect sizes were additionally calculated using Hedges’ g, which incorporates a small-sample correction. Effect sizes were interpreted using conventional thresholds, with values of approximately 0.2, 0.5, and 0.8 corresponding to small, medium, and large effects, respectively, while values ≥ 1.2 and ≥2.0 were considered very large and extremely large effects.

Although the treatment structure contains factorial elements, the present study was designed and analyzed primarily as a seven-group comparative experiment (Control, STZ, STZ + Zn, STZ + DAPA, STZ + DAPA + Zn, STZ + EMPA, and STZ + EMPA + Zn), with n = 5 animals per group. The primary statistical analysis was therefore based on comparisons among the seven experimental groups using one-way ANOVA followed by Tukey’s HSD post hoc test, with non-parametric methods applied where appropriate. The study was not prespecified or powered as a single two-factor factorial experiment for estimating a Drug × Zn interaction.

A formal factorial interaction analysis would require a separate factorial specification for each SGLT2 inhibitor, comparing DAPA ± Zn and EMPA ± Zn within the diabetic groups. Because DAPA and EMPA represent two distinct SGLT2 inhibitor interventions, while the Control and STZ groups serve as reference conditions rather than cells of the same Drug × Zn factorial structure, a single two-way model across all seven groups would not represent the original experimental structure directly. Separate 2 × 2 interaction analyses could be performed as exploratory post hoc analyses; however, these were not part of the prespecified statistical workflow, and the small group size (n = 5) limits the robustness and statistical power of interaction testing. We therefore retained the original group-wise analysis rather than introducing a retrospective factorial analysis.

The factorial nature of the treatment structure was considered when interpreting the combined-treatment groups; however, no formal two-way factorial ANOVA or Drug × Zn interaction analysis was performed. Accordingly, differences observed in the combined-treatment groups are interpreted descriptively as greater or additional treatment-associated effects and are not considered evidence of a statistically demonstrated interaction, additivity, or pharmacological synergy.

Semiquantitative histopathological scores (hepatic injury score and pancreatic islet damage score; see Section 4.4.4) are ordinal variables and were therefore expressed as median [interquartile range] and compared among groups using the Kruskal–Wallis test, followed by Dunn’s post hoc test with Bonferroni correction for pairwise comparisons. Statistical significance was set at *p* < 0.05.

#### 4.4.4. Histopathological and Immunohistochemical Evaluation

At the end of the experimental protocol, blood samples were collected immediately before euthanasia for biochemical analyses. Animals were subsequently euthanized, and liver and pancreatic tissues were carefully excised for histopathological and immunohistochemical evaluation. Tissue specimens were fixed in Bouin’s solution for 24 h, followed by dehydration through a graded ethanol series (70%, 75%, 80%, 85%, 90%, 96%, and absolute ethanol), with each dehydration step lasting approximately 3 h. After dehydration, samples were cleared in three consecutive xylene baths (1 h each) and infiltrated with molten paraffin in three successive baths maintained at 60 °C. Paraffin-embedded tissues were sectioned at 5 μm thickness using a rotary microtome.

For routine histological evaluation, sections were stained with H&E. The staining procedure included deparaffinization, rehydration, hematoxylin staining, eosin counterstaining, dehydration, xylene clearing, and permanent mounting with Canada balsam.

To complement the qualitative histopathological description, H&E-stained liver and pancreatic sections were additionally evaluated using semiquantitative scoring systems. Hepatic sections were scored for macrovesicular and microvesicular steatosis and hepatocellular hypertrophy (0–3 each), according to a rodent-adapted NAFLD scoring system [96], together with a separate necrosis score (0 = absent, 1 = focal, 2 = multifocal, 3 = extensive), as necrotic change is not captured by the original NAFLD Activity Score [97]. Pancreatic islets were evaluated using a 0–4 semiquantitative islet damage score (0 = normal architecture; 1 = mild, <25% of islet area affected by vacuolization; 2 = moderate, 25–50% affected with nuclear pyknosis; 3 = severe, >50% affected with marked reduction in islet size; 4 = very severe, loss of islet architecture), constructed according to general principles for valid histopathological scoring in rodent models. This pancreatic islet damage score was developed for the present study and has not been independently validated; results obtained using this score should therefore be considered exploratory. The principles described by Klopfleisch in his systematic review provide a general framework for histopathological scoring but do not represent independent validation of the specific pancreatic islet damage score developed for the present study [98]. This score has not been independently validated; therefore, results obtained using it should be considered exploratory.

Immunohistochemical analysis was performed to evaluate inflammatory activation, endothelial response, and pancreatic β-cell preservation. The following primary antibodies were used: VCAM-1 (M/K-2; sc-18864), ICAM-1 (G-5; sc-8439), NF-κB (AA143–158; ABIN2627580), and anti-insulin (ABI6939789). Tissue sections were deparaffinized and subjected to heat-induced antigen retrieval in 10 mmol/L citrate buffer (pH 6.0) using microwave treatment at 95 °C for 10 min. After cooling for 20 min, sections were washed twice with phosphate-buffered saline (PBS) and incubated overnight at 4 °C with the respective primary antibodies in a humidified chamber.

Primary antibodies were applied at the following dilutions: VCAM-1 (1:500), ICAM-1 (1:200), NF-κB (1:100), and anti-insulin (1:100). Following PBS washing, sections were incubated with the corresponding horseradish peroxidase-conjugated secondary antibodies. Immunoreactivity was visualized using 3,3′-diaminobenzidine (DAB), followed by hematoxylin counterstaining. Quantitative evaluation of immunohistochemical staining was performed using QuPath software (version 0.5.1, University of Edinburgh, Edinburgh, UK).

Three sections from each animal were analyzed, and digital images were acquired at 400× magnification from five randomly selected fields per section. Detection thresholds were standardized and applied uniformly across all experimental groups. Immunopositive cells were quantified and expressed as the mean number of positive cells/mm^2^. For insulin staining, quantification was performed specifically within pancreatic islets to assess β-cell preservation. The pancreatic assessments were intended to characterize structural preservation of the islets and β-cell insulin immunoreactivity. No circulating endocrine function biomarkers, including fasting insulin or C-peptide, were measured in the present study; consequently, functional indices such as HOMA-IR and HOMA-β were not calculated. Histological scoring and morphometric assessments were performed by the same evaluator according to the predefined assessment criteria described above. The evaluator was not blinded to the experimental group allocation during the assessment. Therefore, the possibility of observer-related bias cannot be completely excluded.

## 5. Conclusions

The present study demonstrates that experimental diabetes induced by the HFD/STZ protocol was associated with substantial hepatic and pancreatic alterations, reflected by increased serum markers of tissue injury, hepatocellular and pancreatic structural damage, enhanced hepatic NF-κB, VCAM-1, and ICAM-1 immunoreactivity, reduced pancreatic islet density and β-cell preservation, and decreased insulin immunoreactivity. Treatment with DAPA or EMPA attenuated several of these diabetes-associated alterations, while the addition of Zn was associated with more pronounced preservation of hepatic and pancreatic tissue integrity, particularly in the EMPA + Zn group.

The integrated biochemical, histopathological, morphometric, and immunohistochemical findings support the relevance of considering the liver–pancreas axis when evaluating tissue protection in experimental diabetes. The results also provide an experimental rationale for further investigating whether Zn supplementation may complement the organ-protective effects associated with SGLT2 inhibition. The additional effects observed with Zn supplementation should be further investigated in specifically designed factorial studies to determine whether they reflect independent additive effects or a true interaction between Zn supplementation and SGLT2 inhibitor therapy. Several limitations should be considered when interpreting the results. The study used an STZ/HFD-induced experimental model and included only male rats, which limits direct extrapolation to the heterogeneous pathophysiology of human T2DM. The relatively small sample size and four-week treatment period may also have limited the detection of moderate treatment effects and the assessment of long-term outcomes. Furthermore, molecular mechanisms underlying the observed changes were not directly investigated, hepatic lipid accumulation was not quantitatively assessed, and pancreatic endocrine function was not evaluated using circulating insulin, C-peptide, or related functional indices. In addition, histological and morphometric assessments were not performed under blinded conditions, and the potential pharmacokinetic or molecular interaction between SGLT2 inhibitors and Zn was not directly examined.

Within these methodological constraints, the present results support further experimental investigation of combined SGLT2 inhibition and Zn supplementation as a potential strategy for limiting diabetes-associated hepatic and pancreatic injury. Future studies using larger cohorts, both sexes, longer treatment periods, metabolically relevant models of T2DM and MASLD, blinded assessment, and direct functional and molecular analyses will be necessary to establish the mechanisms of the observed effects and determine their translational relevance.

## Figures and Tables

**Table 1:** The modification of glycemic/body weight parameters from baseline to endpoint of the experiment.

Group	Body Weight-Baseline	Body Weight-Endpoint	Fasting Blood Glucose-Baseline	Fasting Blood Glucose-Endpoint
Control	242.80 ± 10.64	341.00 ± 75.79	115.20 ± 5.36	113.20 ± 2.17
STZ	238.00 ± 6.48	435.00 ± 8.09	122.60 ± 7.27	475.20 ± 191.55
STZ + Zn	241.40 ± 11.76	367.60 ± 24.87	123.20 ± 12.89	302.00 ± 254.44
STZ + DAPA	234.80 ± 3.49	360.60 ± 34.63	115.60 ± 6.43	143.60 ± 23.90
STZ + DAPA + Zn	247.80 ± 9.52	366.80 ± 66.57	123.20 ± 17.08	150.20 ± 44.90
STZ + EMPA	245.00 ± 10.79	391.80 ± 57.46	123.20 ± 4.09	178.20 ± 142.08
STZ + EMPA + Zn	231.00 ± 8.63	367.20 ± 52.19	129.20 ± 8.04	173.60 ± 140.75

Data are derived from the previously published analysis of the same experimental cohort [60]. HbA1c was not measured in this experimental protocol and therefore could not be included. STZ, streptozotocin; Zn, zinc; DAPA, dapagliflozin; EMPA, empagliflozin.

**Table 2:** Semiquantitative hepatic injury score (median [interquartile range]; steatosis, hepatocellular hypertrophy, and necrosis combined) across experimental groups (n = 5/group). *p*-values from Dunn’s post hoc test with Bonferroni correction, following a significant Kruskal–Wallis test (H = 31.47, df = 6, *p* < 0.001).

Group	N	Hepatic Injury Score Median [IQR]	*p* vs. Control	*p* vs. STZ
Control	5	2 [2-2]	-	0.0002
STZ	5	10 [9-10]	0.0002	-
STZ + Zn	5	9 [9-10]	0.0007	1.0000
STZ + DAPA	5	7 [7-7]	0.1596	1.0000
STZ + DAPA + Zn	5	6 [6-6]	0.9247	0.3262
STZ + EMPA	5	6 [6-7]	0.3867	0.7954
STZ + EMPA + Zn	5	3 [3-4]	1.0000	0.0054

**Table 3:** Semiquantitative pancreatic islet damage score (median [interquartile range]) across experimental groups (n = 5/group). *p*-values from Dunn’s post hoc test with Bonferroni correction, following a significant Kruskal–Wallis test (H = 28.48, df = 6, *p* < 0.001).

Group	N	Pancreatic Islet Damage Score Median [IQR]	*p* vs. Control	*p* vs. STZ
Control	5	0 [0-1]	-	0.0009
STZ	5	4 [4-4]	0.0009	-
STZ + Zn	5	4 [3-4]	0.0031	1.0000
STZ + DAPA	5	3 [3-3]	0.0297	1.0000
STZ + DAPA + Zn	5	3 [2-3]	0.4288	1.0000
STZ + EMPA	5	2 [2-2]	1.0000	0.2219
STZ + EMPA + Zn	5	1 [1-2]	1.0000	0.0238

## Data Availability

The original contributions presented in this study are included in the article/Supplementary Materials. Further inquiries can be directed to the corresponding author.

## Supplementary Materials

The following supporting information can be downloaded at: https://www.mdpi.com/article/10.3390/ijms27209067/s1.
