# Supplementary material for: SGLT2 Inhibition Combined with Zn Supplementation Protects the Liver-Pancreas Axis in Experimental Type 2 Diabetes
Source: Int J Mol Sci. 2026 Oct 13;27(20):9067. doi: 10.3390/ijms27209067 (PMC13705076; doi:10.3390/ijms27209067)
Supplement: Supplementary file 1 [file ijms-27-09067-s001.zip › ijms-4547684-supplementary.pdf]

Supplementary Table S1. Pairwise standardized effect sizes (Hedges' g) for serum ALT and AST activities.

Biomarker	Comparison	Hedges' g	Magnitude
ALT	Control vs. STZ	**5.19**	Extremely large
ALT	STZ vs. STZ+DAPA	**1.44**	Very large
ALT	STZ vs. STZ+EMPA	**1.05**	Large
ALT	STZ vs. STZ+DAPA+Zn	**2.16**	Extremely large
ALT	STZ vs. STZ+EMPA+Zn	**1.71**	Very large
ALT	STZ vs. STZ+Zn	**3.65**	Extremely large
AST	Control vs. STZ	**4.64**	Extremely large
AST	STZ vs. STZ+DAPA	**1.16**	Large
AST	STZ vs. STZ+EMPA	**0.22**	Small
AST	STZ vs. STZ+DAPA+Zn	**0.85**	Large
AST	STZ vs. STZ+EMPA+Zn	**0.31**	Small
AST	STZ vs. STZ+Zn	**4.12**	Extremely large

Abbreviations: Hedges' *g*, standardized mean difference corrected for small sample bias.
Interpretation of effect size: values of approximately 0.2, 0.5, and 0.8 indicate small, medium, and large effects, respectively. Values ≥1.2 were considered very large, whereas values ≥2.0 indicate extremely large effects.

Supplementary Table S2. Pairwise standardized effect sizes (Hedges' g) for serum LDH and ALP activities.

Biomarker	Comparison	Hedges' g	Interpretation
LDH	Control vs. STZ	**14.68**	Extremely large
LDH	STZ vs. STZ+DAPA	**0.38**	Small
LDH	STZ vs. STZ+EMPA	**0.93**	Large
LDH	STZ vs. STZ+DAPA+Zn	**1.51**	Very large
LDH	STZ vs. STZ+EMPA+Zn	**3.39**	Extremely large
LDH	STZ vs. STZ+Zn	**2.40**	Extremely large
ALP	Control vs. STZ	**3.76**	Extremely large
ALP	STZ vs. STZ+DAPA	**2.24**	Extremely large
ALP	STZ vs. STZ+EMPA	**1.52**	Very large
ALP	STZ vs. STZ+DAPA+Zn	**2.13**	Extremely large
ALP	STZ vs. STZ+EMPA+Zn	**1.78**	Very large
ALP	STZ vs. STZ+Zn	**2.78**	Extremely large

Abbreviations: Hedges' *g*, standardized mean difference corrected for small sample bias.
Interpretation of effect size: values of approximately 0.2, 0.5, and 0.8 indicate small, medium, and large effects, respectively. Values ≥1.2 were considered very large, whereas values ≥2.0 indicate extremely large effects.

Supplementary Table S3. Pairwise standardized effect sizes (Hedges' g) for serum total cholesterol and triglyceride concentrations.

Biomarker	Comparison	Hedges' g	Interpretation
Total cholesterol	Control vs. STZ	**1.63**	Very large
Total cholesterol	STZ vs. STZ+DAPA	**1.64**	Very large

Biomarker	Comparison	Hedges' g	Interpretation
Total cholesterol	STZ vs. STZ+EMPA	**0.94**	Large
Total cholesterol	STZ vs. STZ+DAPA+Zn	**0.82**	Large
Total cholesterol	STZ vs. STZ+EMPA+Zn	**0.57**	Moderate
Total cholesterol	STZ vs. STZ+Zn	**0.72**	Moderate–large
Triglycerides	Control vs. STZ	**2.25**	Extremely large
Triglycerides	Control vs. STZ+DAPA	**1.93**	Very large
Triglycerides	Control vs. STZ+EMPA	**2.14**	Extremely large
Triglycerides	STZ vs. STZ+DAPA+Zn	**0.47**	Small–moderate
Triglycerides	STZ vs. STZ+EMPA+Zn	**0.13**	Negligible
Triglycerides	STZ vs. STZ+Zn	**0.59**	Moderate

Abbreviations: Hedges' *g*, standardized mean difference corrected for small sample bias.
Interpretation of effect size: values of approximately 0.2, 0.5, and 0.8 indicate small, medium, and large effects, respectively. Values ≥1.2 were considered very large, whereas values ≥2.0 indicate extremely large effects.
